# High-Accuracy Renal Cell Carcinoma Discrimination through Label-Free SERS of Blood Serum and Multivariate Analysis

**DOI:** 10.3390/bios13080813

**Published:** 2023-08-13

**Authors:** Bogdan Adrian Buhas, Valentin Toma, Nicolae Crisan, Guillaume Ploussard, Teodor Andrei Maghiar, Rareș-Ionuț Știufiuc, Constantin Mihai Lucaciu

**Affiliations:** 1 Department of Urology, La Croix du Sud Hospital, 52 Chemin de Ribaute St., 31130 Quint Fonsegrives, France; buhasbogdan@yahoo.co.uk (B.A.B.); dr.gploussard@gmail.com (G.P.); 2 Department of Urology, Clinical Municipal Hospital, 11 Tabacarilor St., 400139 Cluj-Napoca, Romania; drnicolaecrisan@gmail.com; 3 Faculty of Medicine and Pharmacy, University of Oradea, 1 Universitatii St., 410087 Oradea, Romania; teodormaghiar@yahoo.com; 4 Department of Nanobiophysics, MedFuture Research Center for Advanced Medicine, “Iuliu Hatieganu” University of Medicine and Pharmacy, 4-6 Pasteur St., 400337 Cluj-Napoca, Romania; valentin.toma@umfcluj.ro; 5 Department of Urology, “Iuliu Hatieganu” University of Medicine and Pharmacy, 8 Victor Babes St., 400347 Cluj-Napoca, Romania; 6 Department of Pharmaceutical Physics–Biophysics, Faculty of Pharmacy, “Iuliu Hatieganu” University of Medicine and Pharmacy, 6 Pasteur St., 400349 Cluj-Napoca, Romania

**Keywords:** renal cell carcinoma, human serum, urine, SERS, multivariate analysis, LDA-PCA, SVM

## Abstract

Renal cell carcinoma (RCC) represents the sixth most frequently diagnosed cancer in men and is asymptomatic, being detected mostly incidentally. The apparition of symptoms correlates with advanced disease, aggressive histology, and poor outcomes. The development of the Surface-Enhanced Raman Scattering (SERS) technique opened the way for investigating and detecting small molecules, especially in biological liquids such as serum or blood plasma, urine, saliva, and tears, and was proposed as a simple technique for the diagnosis of various diseases, including cancer. In this study, we investigated the use of serum label-free SERS combined with two multivariate analysis tests: Principal Component Analysis combined with Linear Discriminate Analysis (PCA-LDA) and Supported Vector Machine (SVM) for the discrimination of 50 RCC cancer patients from 45 apparently healthy donors. In the case of LDA-PCA, we obtained a discrimination accuracy of 100% using 12 principal components and a quadratic discrimination function. The accuracy of discrimination between RCC stages was 88%. In the case of the SVM approach, we obtained a training accuracy of 100%, a validation accuracy of 92% for the discrimination between RCC and controls, and an accuracy of 81% for the discrimination between stages. We also performed standard statistical tests aimed at improving the assignment of the SERS vibration bands, which, according to our data, are mainly due to purinic metabolites (uric acid and hypoxanthine). Moreover, our results using these assignments and Student’s *t*-test suggest that the main differences in the SERS spectra of RCC patients are due to an increase in the uric acid concentration (a conclusion in agreement with recent literature), while the hypoxanthine concentration is not statistically different between the two groups. Our results demonstrate that label-free SERS combined with chemometrics holds great promise for non-invasive and early detection of RCC. However, more studies are needed to validate this approach, especially when combined with other urological diseases.

## 1. Introduction

Worldwide, renal cell carcinoma (RCC) represents the 6th most frequently diagnosed cancer in men and the 10th in women, accounting for 5% and 3% of all cancers, respectively [1,2]. RCC incidence rates have been increasing annually during the last two decades until recently, with about 2% in incidence both worldwide and in Europe, leading to approximately 99,200 new RCC cases and 39,100 kidney cancer-related deaths within the European Union in 2018 [1,2].

Many renal masses remain asymptomatic until the late disease stages. Most RCCs are detected incidentally by non-invasive imaging investigating various non-specific symptoms and other abdominal diseases [3]. RCC is the most common solid lesion within the kidney, accounting for approximately 90% of all kidney malignancies [4]. 

In a recent multicenter prospective observational cohort study, 60% of patients overall, 87% of patients with stage 1a renal tumors, and 36% of patients with stage III or IV disease presented incidentally [5].

There is a 1.5:1 predominance of men over women, with a higher incidence in the older population [2,6]. RCC comprises a broad spectrum of histopathological entities described in the 2022 World Health Organization (WHO) classification [4]. There are three main RCC types: clear cell (ccRCC), chromophobe (chRCC), and papillar (pRCC). In general, ccRCC has a worse prognosis compared to chRCC and pRCC [7,8]. 

The well-known triad of visible hematuria, flank pain, and palpable abdominal mass is rare (6–10%) and correlates with advanced disease, aggressive histology, and poorer outcomes [5,9,10].

Renal tumors are diagnosed by abdominal ultrasound or computed tomography performed for other medical reasons and are classified as solid or cystic based on imaging findings [11]. The most important criterion for differentiating malignant lesions is the presence of contrast enhancement [12]. Despite having good sensitivity and specificity, contrast-enhanced computer tomography (CECT) is unsuitable for screening because of its high irradiation, invasive nature, and lack of cost-effectiveness [7]. Ultrasonography has emerged as a potential screening tool, but its accuracy is influenced by tumor size and location and is operator-dependent [8]. Several serum and urine biomarkers have been studied, but none of them have yet been validated [9,10,11]. In recent years, there is a growing interest from both patients and clinicians in RCC screening programs; however, there is a relative lack of studies reporting the efficacy, cost-effectiveness, and optimal modality for RCC screening [12,13,14]. Body fluids such as blood, urine, saliva, cerebrospinal fluid, or breast milk can non-invasively reflect the condition of affected body tissues. This paves the way for an effective alternative to traditional tissue biopsies. Liquid biopsies offer faster results; they can help predict metastasis risk and monitor the progression of cancer more regularly, inexpensively, and efficiently. Various substances within these fluids, capable of conveying cancer-related information, are discharged, making them excellent potential biomarkers. These include proteins or peptides, genetic material, or circulating tumor cells (CTCs) that have detached from the tissues. Additionally, microRNAs (miRNAs) have gained attention as cancer biomarkers due to their proven role in tumor progression and metastasis. Similarly, examining circulating tumor DNA (ctDNA) for significant somatic mutations (via liquid biopsy) is seen as a major breakthrough in precision oncology for Renal Cell Carcinoma (RCC), facilitating real-time tracking of the disease’s molecular progression. Nonetheless, our understanding of ctDNA analysis’s utility in RCC is still limited, partially due to the absence of assays specifically designed for ctDNA analysis in RCC, although some progress in this field was reported recently [15]. However, the European Association of Urology 2023 Guidelines on RCC state there are no clinically validated urinary or serum biomarkers that have yet been identified, and no established screening tool currently exists for RCC [16]. 

In the last decades, vibrational spectroscopy techniques such as infrared and Raman spectroscopy, through many proof-of-concept studies, have been revealed as methods with huge potential in the field of medical diagnosis [17]. The molecular vibration spectra of biological tissues or liquids contain important information related to their biochemical composition, which in principle can be used to discriminate certain pathological conditions, including cancer. However, the Raman and infrared spectra of biological materials are dominated by their major components (proteins and lipids), masking in many cases the signals of specific markers for pathological conditions, which in most cases have low concentrations and much lower molecular masses. The development of the Surface-Enhanced Raman Scattering (SERS) technique, which uses either gold (AuNPs) or silver nanoparticles (AgNPs) to amplify the Raman signals of molecules that attach to metal surfaces, opened the way for investigating and detecting small molecules, especially in biological fluids. The SERS investigation of biological liquids such as serum or blood plasma, urine, saliva, and tears (the so-called liquid biopsy) presents major advantages from the point of view of their noninvasive collection, the small amounts of samples required, the non-destructive nature of the investigation, and the openness of the patients. It was proposed as a simple technique for the diagnosis of various diseases, including cancer [18].

The SERS spectra are strongly Influenced by the affinity between the SERS substrate (gold or silver nanoparticles) and the investigated molecules, as well as by the orientation of the molecules on the surface, but with a judicious choice of the SERS substrates and their appropriate functionalization, unparalleled performances were achieved in the detection of molecules of biological interest (unimolecular detection) [19]. The main impediment to the use of the SERS technique in the biomedical field is primarily related to the low reproducibility of the spectra, which depends, among other things, on the nature of the substrate used (colloid or solid substrate, nature of the metal, surface coating), nature of the sample used (solid/dry or liquid), the ionic composition of the medium, and the wavelength of laser radiation used for Raman excitation. In the case of blood plasma or serum, it was observed that the SERS spectra are dominated by proteins due to NP opsonization [20]. Therefore, the detection of smaller molecular mass components needs the previous deproteinization of the sample, either by filtration [20] or by chemical means [21]. The pioneering work by Premasiri et al. [22] showed for the first time that using solid SERS substrates with blood components allows evidence of low molecular compounds, with the SERS spectra being dominated by purine metabolites (uric acid and hypoxanthine) without the need for deproteinization. Our research group previously reported that a solid plasmonic SERS substrate, synthesized using purified and concentrated hydroxylamine-reduced AgNPs, through a Tangential Flow Filtration (TFF) technique, produces reproducible SERS spectra able to discriminate breast cancer patients through label-free SERS (LF-SERS) of blood plasma combined with multivariate analysis (MVA), with relatively high accuracy [23]. 

The SERS profiling of serum for RCC diagnosis in a limited number of patients was reported recently [24] with an average accuracy of 0.77 based on three different machine learning algorithms. Bai et al. [25] used SERS of blood serum and 3 multivariate analysis algorithms for the discrimination of 38 kidney and 26 bladder cancer patients from 39 healthy donors and reported that the best discrimination accuracy was obtained by a Supported Vector Machine (SVM)-based algorithm. In this paper, we propose to increase the accuracy of the RCC discrimination and improve the interpretation of the obtained results. In this sense, we increased the number of patients included in the study (50 RCC male patients and 45 controls) and used simple statistical tests to improve the assignment of the major vibrational bands, aiming to gain more insights into the key factors that influence discrimination accuracy. Two multivariate analysis tests—Principal Component Analysis combined with Linear Discriminate Analysis (PCA-LDA) and Supported Vector Machine (SVM)—were used for the discrimination of cancer patients from controls. We also demonstrate how different parameters used in the statistical analysis could improve the discrimination accuracy of the SERS data. It is important to acknowledge that even if these algorithms function by providing high discrimination accuracy (usually >90%), a precise interpretation of LF-SERS data and knowledge of the molecular origin of each band from a biochemical perspective can greatly aid in evaluating the credibility of the results. Additionally, a correct assignment of these vibration bands has the potential to enhance our understanding of a particular disease from a biochemical standpoint, thereby suggesting new avenues for further studies and research. 

## 2. Materials and Methods

Silver nitrate (AgNO_3_) and hydroxylamine (NH_2_OH–HCl) from Roth GmbH (Gladbach, Germany) were of analytical grade and were used as received. CaF_2_ polished glasses (Crystran Limited, Poole, UK) with one single Raman peak at 321 cm^−^^1^ supported the solid substrates.

### 2.1. Synthesis of the Substrate

The solid plasmonic substrates were prepared using an original method developed by our group at the MEDFUTURE Research Center for Advanced Medicine, University of Medicine and Pharmacy Cluj-Napoca [23]. The AgNPs were prepared by reducing silver nitrate with hydroxylamine, according to the procedure proposed by Leopold and Lendl [26]. Briefly, 12 mg of NaOH and 10.4 mg of NH_2_OH HCl were dissolved in 90 mL of deionized water under stirring (500 rpm). To this solution, 10 mL of a solution containing 17 mg of AgNO_3_ was added quickly. The mixture turned brown immediately and, after a few minutes, became yellowish. The absorption spectrum of the colloid obtained presents a maximum of around 408 nm. The silver colloids were purified and concentrated to 10× by using a tangential flow filtration (TFF, Pall Corporation, New York, NY, USA) technique, as described previously [23,27]. This filtration step leads to a reduced polydispersity of silver colloids and removes the byproducts resulting from synthesis, increasing their SERS performance [28]. The CaF_2_ glass was cleaned using acetone and ethanol, then rinsed with ultrapure water before being left to air dry. After 15 min, the port-probe was heated to 40 °C using a plate heater. Subsequently, 1 microliter of concentrated colloid was applied to the CaF2 port probe and allowed to dry for 80 **s**. Once the SERS solid substrates were prepared, they were taken off the heated plate to cool to room temperature, at which point they were ready for use. This type of SERS substrate can provide reproducible SERS spectra of biological fluids or other analytes, as previously demonstrated [23,27]. 

### 2.2. Analyte Deposition and SERS Measurements

A volume of 1 μL of blood serum was pipetted onto the dry solid substrates and allowed to dry at room temperature before conducting SERS measurements. The SERS spectra were acquired using a Renishaw inVia Reflex Raman confocal multilaser spectrometer (Renishaw plc, Gloucestershire, UK) at a resolution of approximately 2 cm^−^^1^. The wavenumber calibration was carried out using an internal silicon reference. A laser excitation wavelength of 785 nm was used. The laser beam was directed to the sample through a 50× objective lens (N.A. = 0.75) of a Leica microscope. The spectra were recorded using a 600 lines/mm grating and a charge-coupled device (CCD) camera. The laser power was set at 1% of the maximal power and was measured to be 2.2 mW at the sample for the above optical configuration. The laser beam was focused at a distance of approximately 40–50 μm from the edge of the dried sample, and the spectrometer was set to collect data in mapping mode. The acquisition time was set to 10 s, and a total of 50 spectra from different points were acquired. Two maps, each consisting of 50 points, were recorded for each sample. The Wire 4.2 software provided by Renishaw (Gloucestershire, UK) in conjunction with the inVia spectrometer was utilized to perform baseline correction.

### 2.3. Research Ethics

All subjects gave informed written consent for inclusion before participating in the study. The study was conducted according to the Declaration of Helsinki 2013, and the protocol was approved by Cluj-Napoca Municipal Clinical Hospital, Ethics Committee Decision No. 1 from 19 January 2018 for the study entitled “Biomarkers for early diagnosis of bladder, prostate, and kidney cancer by Raman spectrophotometric profile analysis of biological fluids (blood and urine) and humoral tissues involving humans”.

### 2.4. Cohort of Patient Samples

A total of 118 samples from patients diagnosed with renal tumors were collected after CECT with informed written consent. A total of 50 male RCC patients and 45 healthy donors were enrolled in the study. The samples for RCC patients were collected before surgery or any other therapy. The diagnosis was confirmed after surgery by histopathology. To reduce sample variability, only male subjects were included in the study. In the case of cancer patients, only those diagnosed with clear-cell RCC were included. The patient ages ranged between 38 and 78 years, with a median of 63.5 years and a standard deviation of 9.8 years. In the case of healthy donors, the ages ranged between 19 and 88 years, with a median of 62 years and a standard deviation of 16.4 years. More information about the cohort of patients and their histopathological profiles is provided in Tables S1 and S2. 

### 2.5. Blood Serum Collection

After overnight fasting, blood samples were obtained from the antecubital vein of healthy donors and RCC patients using Becton Dickinson vacutainers specially designed for serum. After collection, blood samples were allowed to clot for 30 min and centrifuged at 3000× *g* for 10 min to separate the serum from the rest of the blood. The serum was collected through a pipette, transferred to special containers to be frozen, and kept at −80 °C until SERS analysis. 

### 2.6. Multivariate Analysis

The multivariate analysis (MVA) of the experimental SERS spectra was performed using the Unscrambler X 10.5.1. Licensed Software package (Camo Analytics, Oslo, Norway). All the experimental data have been baseline corrected using the Wire software. The other data preprocessing steps, as detailed in the Results section, were performed using the same MVA software. The OriginPro 2016 (OriginLab, Northampton, MA, USA) software was also used for the graphical representation of the data and simple statistics.

## 3. Results and Discussion

### 3.1. SERS of Blood Serum Samples

#### 3.1.1. Major SERS Vibrational Peaks in the Serum Samples and Their Tentative Assignment

**Figure 1:**
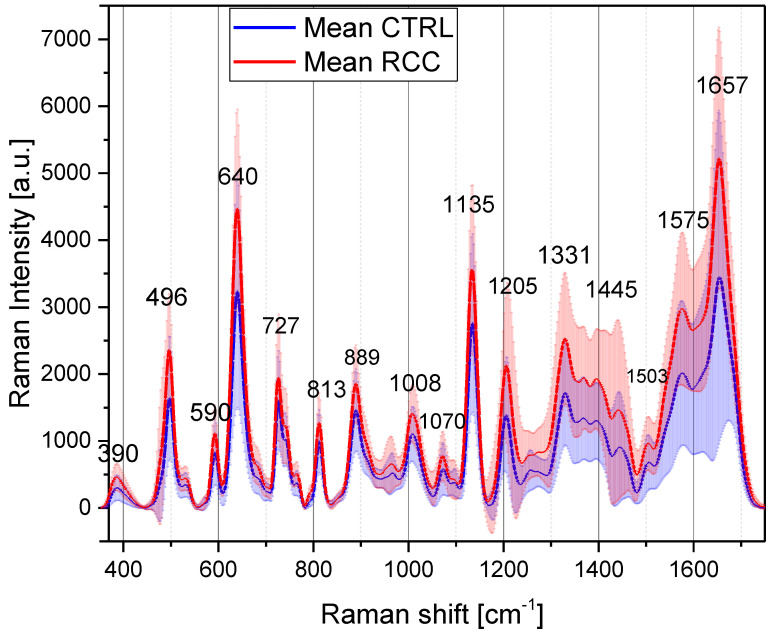
Mean SERS spectra of serum samples collected from controls (blue) and RCC (red) their standard deviations and the main vibrational peaks.

In this paper, each serum sample was dried on the substrate, and the SERS spectra were recorded at 50 different points on the dried serum droplet using the Raman spectrometer in the mapping mode. Two maps of 50 points each were averaged so that each sample spectrum represents the average of 100 SERS spectra. The maps were located around 50 µm from the dried droplet edge, in areas of uniform thickness. The mean spectra recorded on cancer samples (red spectrum) and controls (blue spectrum) are presented in Figure 1. Typical SERS spectra of serum samples for both controls and RCC patients are presented in Figure S1A. The main vibration peaks, their corresponding Raman shifts, and their assignments according to the literature data and the assignments proposed in this study are provided in Table 1. Please note that in the literature there are slight differences in the wavenumbers of the SERS peaks reported, and therefore in the second column of Table 1, we provided ranges instead of fixed wavenumbers. We also calculated the mean SERS intensities for the two maps and obtained good overlapping between the two sets of data (Figure S1B), proving the SERS substrate’s quality. 

The blood serum is a complex fluid containing, proteins, lipoprotein aggregates, hormones, vitamins, nucleic acids, and metabolites. In the label-free approach, the SERS spectra contain information mainly about the molecules for which the Raman signals are significantly amplified by the metal surfaces. Therefore, the label-free SERS spectra of serum strongly depend mainly on molecules’ affinity for plasmonic substrate and, to a lesser extent, on their relative concentration. Another factor that strongly affects the SERS spectra of blood serum or plasma is the nature of the substrate and its coating. In the case of colloidal silver and gold nanoparticles used with serum or plasma, the nanoparticles will be surrounded by proteins that will dominate the SERS spectra [20,22]. Smaller molecular mass species can be evidenced by SERS only by sample deproteinization using either chemical (e.g., methanol) or physical (ultrafiltration) techniques. On the other hand, as these colloids are prepared through wet chemical techniques, in most common synthesis methods, the NPs are negatively charged, and therefore, they will amplify the signal of cationic analytes. Special synthesis techniques were proposed for the detection of anionic analytes (nucleosides and DNA fragments) [41].

The use of solid plasmonic substrates offers a potential solution to overcome many of these limitations. Research has shown that when employing these substrates with a near-infrared excitation wavelength of 785 nm, the most prominent bands observed in SERS mainly correspond to molecules associated with purine metabolism (such as uric acid, hypoxanthine, and xanthine), glutathione, and proteins (in the case of unfiltered, whole biofluids) [20,22,31,32,33,34,35,36,37,38,39,40]. More recently, the presence of ergothioneine (ET), a dietary amino acid with a speculated vitamin-like role, has also been detected using SERS in biofluids [39,42]. Furthermore, it is worth noting that the use of lower wavelength laser excitations (532 nm and/or 633 nm) primarily leads to the occurrence of vibrational bands specific to carotenoids. This phenomenon arises from a resonant Raman effect [20,21,24].

The assignment of SERS vibrational bands in blood serum or plasma lacks consensus within the SERS community. Table 1 clearly illustrates that different molecular species and sometimes even different vibration modes are assigned to the same experimentally observed Raman shift wavenumber. These inconsistencies primarily arise from assigning SERS bands to wavenumbers observed in the Raman spectra of biological molecules without clear experimental evidence. Only a few papers compare the SERS signals of blood serum or plasma with those of relevant molecules obtained under the same experimental conditions. These conditions include the use of the same substrate, excitation wavelength, and ionic composition of the medium, among others [20,22,39,42], and our tentative assignments are based on these references together with combined experimental and theoretical papers related to SERS of uric acid [43] and hypoxanthine [44], as will be detailed further.

As can be seen from Figure 1, the most intense vibrational bands in the spectra of blood serum are characterized by a Raman shift of 640 cm^−1^, 1135 cm^−1^, assigned to uric acid [20,22], and 1657 cm^−1^, assigned to amid I vibration of proteins (Table 1). Another intense peak can be noticed at 727 cm^−1^. Premasiri et al. [22] showed that it can be assigned to hypoxanthine and that its intensity increases with the time of the blood sample “aging”, i.e., the time between the blood collection and the separation of the cells through centrifugation, leading to a SERS spectrum that is fully dominated by hypoxanthine after several hours of “aging”. For this reason, we proceeded with the separation of the cell from the blood no later than 30 min after the blood collection. 

Other bands present in the SERS signals of serum occur at 590 cm^−1^, 811 cm^−1^, 889 cm^−1^, and 1205 cm^−1^ and could be also assigned to uric acid according to the literature (Table 1). A combined experimental and Density Functional Theory (DFT) study [43] evidenced the above-mentioned vibrational bands as characteristic of uric acid. The 640 cm^−1^ peak is associated with uric acid skeletal ring deformation, while the 1135 cm^−1^ vibration band is associated with its CN stretching [43]. In the case of hypoxanthine, the SERS spectrum collected on the solid substrate is dominated by an intense band at 728 cm^−1^, which the DFT calculation assigned to the purine breathing mode [44] and which matches the intense vibration measured for our serum samples at almost the same wavenumber.

Another important feature of the SERS spectra of serum is the relatively intense peak recorded at 496 cm^−1^ which might be assigned to uric acid [43]. However, recently, an intense SERS peak for blood components (serum plasma or red blood cell lysates) recorded around 484 cm^−1^ was assigned to ergothioneine, a vitamin-like amino acid [39,42]. It was also claimed [42] that several other peaks in the SERS profiles of biological fluids are due to this dietary amino acid, in the wavenumber ranges 1124–1132 cm^−1^, 1214–1224 cm^−1^, 1310–1323 cm^−1^, 1442–1445 cm^−1^ and 1575–1582 cm^−1^. The latter two vibrational bands are also superposed over the vibrational bands assigned to hypoxanthine [20,22]. 

#### 3.1.2. Correlations between the SERS Intensities of Major Vibrational Peaks

Aiming to gain more insights into the assignment of the serum samples’ bands, we performed a linear regression analysis of the different SERS peak intensities, calculating the coefficient of determination for the corresponding linear regression. The correlation curves and the coefficients of determination are provided in Figures S2–S4 for 640 cm^−1^, 727 cm^−1^, and 484 cm^−1^ bands, respectively.

The synthetic data for the correlations of the SERS intensity at 640 cm^−1^ (assigned to uric acid) and 727 cm^−1^ (assigned to hypoxanthine) with other major vibrations are presented in Table 2 and for the 484 cm^−1^ vibrational band in Table S3.

As can be seen from Table 2, the SERS intensity at 640 cm^−1^ correlates with a coefficient of determination higher than 0.9 with the SERS intensities at 496 cm^−1^, 590 cm^−1^, 765 cm^−1^ (shoulder), 812 cm^−1^, 889 cm^−1^, and 1135 cm^−1^. This observation supports the idea that the SERS signal recorded at these wavenumbers could be assigned to uric acid, as these vibration bands occur also in the SERS spectrum of pure uric acid [20,22]. Furthermore, the coefficients of determination for the linear correlations of the SERS intensities corresponding to the above-mentioned vibration bands are greater than 0.9 (Figure S2), thus supporting the idea that they belong to the same molecular species. We acknowledge that high values of the coefficients of determination for the linear correlation between two bands’ intensity are not unequivocal proofs that the two bands are signals from the same molecular species. It is also possible that the molecular species responsible for the two vibrations have the same change from one sample to another, or, in other words, that their concentrations in different samples change similarly.

Other vibrations featured in the uric acid SERS spectrum with coefficients of determination in the 0.8–0.9 range occur at 390 cm^−1^, 531 cm^−1^ (shoulder), 1008 cm^−1^, 1070 cm^−1^, 1503 cm^−1^, 1575 cm^−1^ and 1657 cm^−1^ (Table 2). In the latter cases, the lower coefficient of determination values might be explained by the fact that we might have a superposition at these wavenumbers with the vibration of other molecules. 

Concerning the peaks recorded at 1575 cm^−1^ and 1657 cm^−1^, it was experimentally proven [20] that their intensity strongly decreases for deproteinized serum or plasma samples, and, as a consequence, it was suggested that, at least partly, these vibrations are assignable to proteins (amid II and I, respectively). Other interesting observations are related to the peaks recorded at 1330 cm^−1^, 1369 cm^−1^, and 1445 cm^−1^ which all strongly increase their relative SERS intensities in the case of deproteinized samples [20] indicating that they are related to low molecular mass compounds. The 1445 cm^−1^ peak was initially assigned to CH_2_/CH_3_ bending vibration of lipids [20,35], but also to hypoxanthine [20,22]. Ergotheonine also presents specific bands at this wavenumber [39,42].

In the case of the 727 cm^−1^ vibration (hypoxanthine), no high coefficient of determination correlation could be established between the SERS intensity at this wavenumber and the SERS intensity of other peaks (Table 2). This result suggests that the only peak assignable to hypoxanthine, based on this type of correlation, is at 727 cm^−1^. Concerning the 484 cm^−1^ wavenumber, which was suggested to be assigned to ergothioneine [42], we noticed first that for the mediated spectra (both from controls and RCC) we recorded a maximum SERS intensity at 496 cm^−1^ which strongly correlates with other peaks assigned to uric acid (Table 2). On the other hand, no significant high coefficients of determination were found with other peaks observed in the SERS of serum. We also noticed a high correlation between the SERS intensity at 484 cm^−1^ and the SERS intensity at 1221 cm^−1^ (R^2^ = 0.97) (Table S3), which was reported to be a characteristic peak of ergothioneine [39,42]. The SERS peak recorded in serum samples is close to this wavenumber at 1205 cm^−1^ and was not assigned to uric acid or hypoxanthine. Therefore, we cannot exclude that ergothioneine has a contribution to this maximum, but at 484 cm^−1^ the peak of ergothioneine is most probably masked by uric acid. Looking closer at all the serum spectra recorded in this low wavenumber range (Figure S5), we noticed that some samples present a maximum at 480 cm^−1^, most of the spectra have a maximum of 496 cm^−1^, among the latter, some samples show a shoulder around 484 cm^−1^. Therefore, these results suggest that both molecules (ergothioneine and uric acid) present SERS peaks in this narrow wavenumber range, but when the spectra are mediated, the 496 cm^−1^ peak of uric acid dominates in most of the samples. Our results also suggest that the maximum in the SERS of ergothioneine occurs at 480 cm^−1^ instead of 484 cm^−1^, at least for our substrate and excitation conditions. These very slight differences in the vibration frequencies between uric acid and ergothioneine could be easily understood by the structural similarities of the two molecules, which share a common heterocycle. Recently, it was claimed that apart from the 484 cm^−1^ band, other peaks that are commonly detected in blood serum (1131 cm^−1^, 1215 cm^−1^, 1445 cm^−1^, 1575 cm^−1^) could be also assigned to ergothioneine [39,42]. It was also hypothesized that the thiolate form of ergothioneine is adsorbed on the metal surface via the S and N atoms of its heterocyclic moiety [42]. Nevertheless, theoretical studies at the DFT level of theory, specifically dedicated to ergothioneine and a comparison with uric acid, are needed for a clear-cut answer regarding the assignments of the vibration bands shared by the two compounds. We also noticed a slight correlation (R^2^ = 0.75) of the SERS intensity at 484 cm^−1^ with the peak at 1445 cm^−1^, implying that ergothioneine might have contributed to the SERS signal recorded at this wavenumber.

Based on these general observations, it can be concluded that when using an excitation wavelength of 785 nm, the dominant molecules contributing to SERS signals in serum are uric acid, hypoxanthine, and potentially ergothioneine. The surface of the substrate appears to function as a “spectroscopic filter”, selectively enhancing the signals of these particular metabolites, despite the presence of over 4000 identified metabolites in human blood serum [39].

Another interesting feature of the serum SERS is the peak at 2107 cm^−1^ assigned to stretching the CN triple bond within the thiocyanate group (SCN). Recently, it was demonstrated that this peak is relevant in saliva samples and is more intense in patients submitted to low-dose dental irradiation [45]. While its intensity is very small and can be identified only in some samples in the case of unfiltered serum, its relative intensity significantly increases after the serum samples are filtered by centrifugation through 3 kDa filters. 

Although we recorded the SERS spectra in the range 350–2500 cm^−1^, because the signal at 2107 cm^−1^ was too small, in further analysis, we considered only the spectrum range 353–1750 cm^−1^, as the SERS signals above 1750 cm^−1^ do not improve the discrimination. On the contrary, extending the Raman shift range above 1750 cm^−1^ induces “noise”, decreasing the discrimination accuracy.

Regarding 1575 cm^−1^ and 1657 cm^−1^ peaks, it was experimentally proven [20] that their intensity strongly decreases for filtered serum or plasma samples, and, as a consequence, it was suggested that, at least partly, these vibrations are assignable to proteins (amid II and amid I, respectively). Other interesting observations are related to the peaks recorded at 1330 cm^−1^, 1369 cm^−1^, and 1445 cm^−1^, which all increase strongly for filtered samples [20] indicating that they are related to low molecular mass compounds. The 1445 cm^−1^ peak was initially assigned to CH_2_/CH_3_ bending vibration of lipids [20,35], but also to hypoxanthine [20,22]. Ergotheonine also exhibits vibration bands at this wavenumber [39,42].

### 3.2. Descriptive Statistics

As a general observation, one can notice that the peak intensity is increased in the case of RCC serum samples as compared to healthy donor ones. It should be noted that the Raman intensities in Figure 1 are normalized to the laser power and the acquisition time. Corroborating this observation with the fact that most vibrational bands could be assigned to products of purinic metabolism (e.g., uric acid, hypoxanthine), one can state that the serum samples obtained from RCC patients have a higher concentration of some of these molecules. For a quantitative estimation of the differences between the RCC patients and the healthy donors, we performed a statistical significance test for the major vibration bands using the Student’s *t*-test. The main results of the test, performed with the Unscrambler program level for a 95% confidence level (described as α = 0.05), are summarized in Table 3, and two examples for the vibration bands assigned to uric acid and hypoxanthine are provided in Figure S6.

There are many peaks for which the SERS intensities measured in the case of patients are significantly higher as compared to healthy donors. This type of result is seen for all the peaks assigned to uric acid, but also for those related to proteins (1575 cm^−1^, 1657 cm^−^^1^) and peaks that were not clearly assigned (1205 cm^−1^, 1331 cm^−1^, 1445 cm^−1^). A major exception was, however, recorded for the peak at 727 cm^−^^1^ assigned to hypoxanthine, for which no significant difference between the controls and RCC patients can be detected. The same increase in the SERS signals of uric acid bands [24] and especially for the peak at 640 cm^−^^1^ [25] was found for kidney cancer cases compared with apparently healthy donors, our results being in agreement with previous reports.

Uric acid (UA) is the final product of purine catabolism in humans and great apes, and its concentration is maintained at high blood levels through filtration and reabsorption [46]. High levels of uric acid (>360 µM) are found in hyperuricemia, and even higher levels (>700 µM) are usually associated with gout and acute kidney injury. A correlation between high levels of serum uric acid and obesity, metabolic syndrome, diabetes, and inflammation has been described by different authors, and some types of cancer, including kidney cancer, appear to be strongly connected with inflammation, metabolic syndrome, and obesity [47]. The enzyme responsible for the endogenous production of UA is xanthine oxidoreductase (XOR), which catalyzes the last two irreversible steps of purine catabolism, the oxidation of hypoxanthine to xanthine and of xanthine to uric acid. The involvement of XOR in cancer was consistently investigated, and its expression and activity appear variable in different types of tumors, where the enzyme has been shown to play either suppressive or oncogenic roles [48,49]. On the other hand, the serum acid level also depends on its excretion from the body, 70% of which occurs at the kidney level. In a recent epidemiological study on over 0.44 million participants, it was found that serum uric acid levels were positively associated with the risk of kidney cancer [50]. Similar results have been found in many retrospective and prospective studies of nonurological malignancies. 

The precise mechanism linking elevated levels of serum uric acid (SUA) and kidney cancer remains uncertain. One potential explanation is impaired renal excretion as well as the high cell turnover observed in cancer cells. Another potential factor is the increased activity of xanthine oxidase, leading to heightened purine metabolism and subsequently elevated SUA levels. This rise in SUA is frequently observed during apoptosis, a process associated with cancer progression and cell renewal. Additionally, increased oxidative reactions triggered by the tumor may contribute to higher levels of SUA [50].

Our results, showing an increased concentration of uric acid in patients with RCC as compared to healthy donors, are in agreement with the above-mentioned findings. Moreover, the fact that hypoxanthine levels are comparable in RCC patients with healthy donors suggests that the increased levels of SUA in RCC are most probably due to an impaired renal excretion mechanism rather than an increased production, which is also plausible as the kidney functions might be affected by the disease. However, we believe that a full and correct assignment of the SERS vibration bands of serum based on both theoretical and experimental studies will shed more light on the changes observed in pathologies.

### 3.3. Multivariate Analysis

#### 3.3.1. Principal Component Analysis

The univariate analysis of spectroscopic data has limited applicability in the study of biological fluids due to their complex composition, and multivariate techniques have been used extensively in the last few decades in this field.

In the first step, we proceeded with an unsupervised Principal Component Analysis, aiming to reduce the dimensionality of the data. Each spectrum contains 1015 data points in the wavenumber range 353–2533 cm^−^^1^. We reduced this spectral range to 353–1750 cm^−^^1^ as no significant features were recorded above 1750 cm^−^^1^, as mentioned above. Therefore, each spectrum contains 605 data points. PCA is a valuable tool for exploratory data analysis as it reduces the dimensionality of large data sets, provides a visual representation of sample relationships, and helps identify influential variables. It aids in revealing hidden structures and patterns within the data, thereby enabling us to gain insights into the underlying mechanisms or factors that drive similarities or differences among samples.

**Figure 2:**
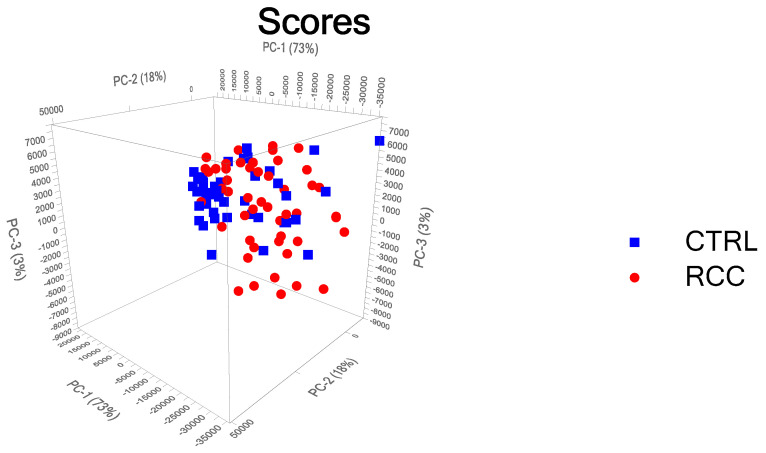
Score plot for the PCA analysis for the first 3 PCs, which explains 99% of the variance.

We chose to reduce the dimensionality to 12 Principal Components (PC), which account for more than 99% of the variance in the data. The score plot for the first 3 PCs is provided in Figure 2, while the explained variance is provided in Figure S7, and the loading curves for PC 2 and PC 12 are given in Figures S8 and S9, respectively.

**Figure 3:**
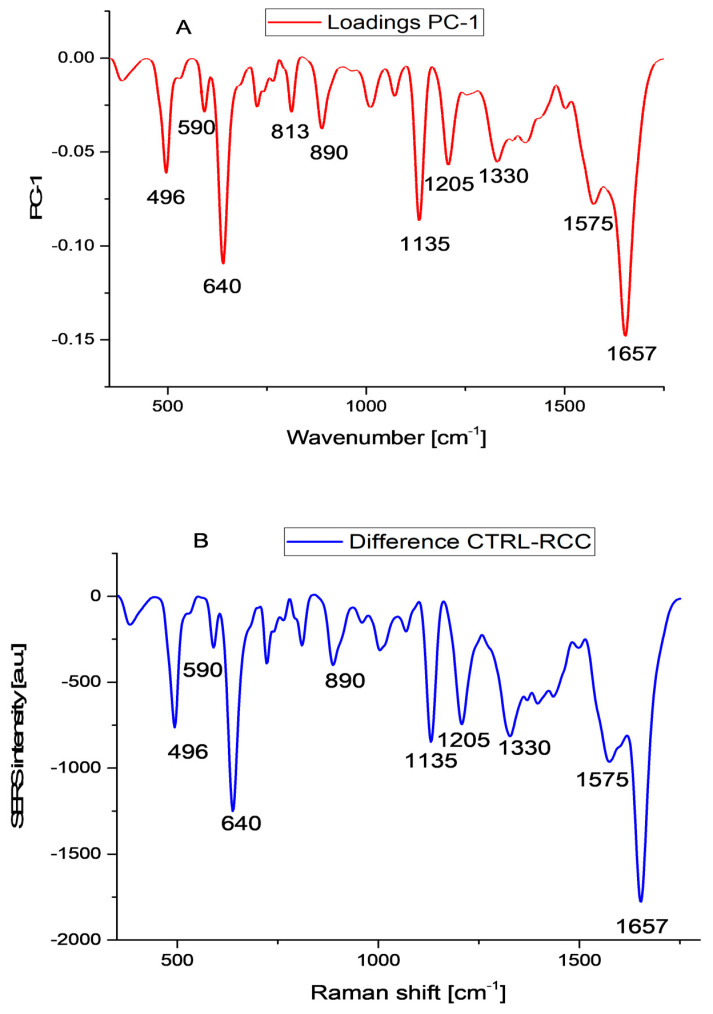
(**A**) Loadings plot for the first principal component PC1 and (**B**) the difference spectrum between the mean SERS of control and RCC patients samples.

For the validation of the model, we selected the leave-one-out cross-validation (LOOCV) method, which means that one sample was compared with all the other samples. As can be seen from Figure S7, the first principal component explains 73% of the variance and the second one another 18%, meaning that the first two components explain 91% of the variance. The loading plots show that PC1 (Figure 3A) has several negative peaks at 496 cm^−1^, 640 cm^−1^, and 1135 cm^−1^, which were assigned to uric acid, and 1657 cm^−1^, assigned to amide I in α-helix proteins, which are also the major peaks present in the difference plot between the controls and RCC cases (Figure 3B). The loading plot for PC2 (Figure S8) is characterized by peaks at 490 cm^−1^, 1215 cm^−1^, and 1445 cm^−1^, which might be related to ergothioneine.

#### 3.3.2. Linear Discriminant Analysis on Raw Data

**Figure 4:**
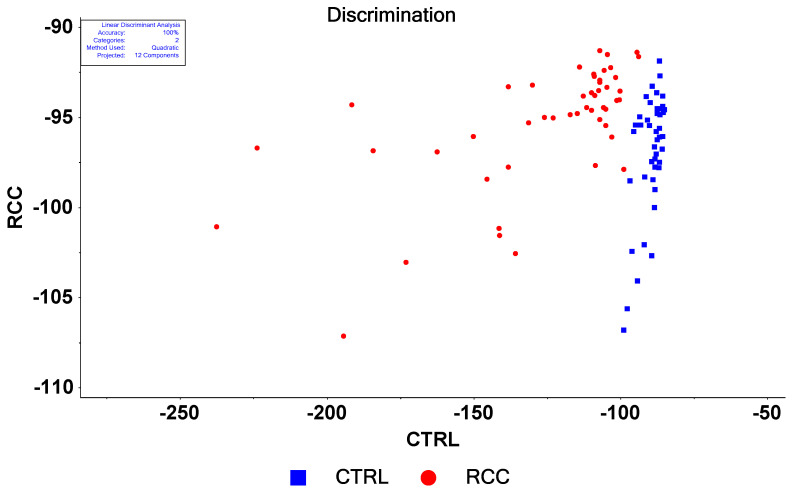
Discrimination plot obtained by using a quadratic discrimination function, 12 Principal Components.

In the next step, we applied a supervised classification method, aiming to separate the two sets of data. As such, linear discrimination analysis was combined with principal component analysis (LDA-PCA). The results obtained by considering 12 PCs and using a quadratic discrimination function are presented in Figure 4. The samples are classified based on their scores using the same LOOCV, but this time in the supervised model: one sample was compared with all the others by knowing the category to which all the other samples belong. For the parameters indicated above, we obtained an accuracy of discrimination of 100%.

We systematically investigated how the number of PCs and the discrimination function influence discrimination accuracy, and the results are presented in Table S4.

From the above data, it seems that the quadratic discrimination function yields the highest accuracy in the discrimination between controls and RCC patients, followed by the Mahalanobis function. The linear discrimination function gives the worst accuracies. It is interesting to note that the Mahalanobis function provides the best sensitivity (100% at 10 components; 98% at 8 and 9 components; 94% at 5 components). High specificity is extremely important for the medical screening tests in practice because no other investigations are performed for misclassified false negative cases unless specific symptoms are reported (usually in a more advanced stage of the disease with a much less positive outcome). However, if the high specificity is not correlated with a high sensitivity, the usefulness of the test is significantly reduced as the false positive cases involve further investigations, increasing significantly the medical costs involved.

It is obvious that by increasing the number of components, the discrimination accuracy increases, but much care should be taken not to overfit the data. There is no definite, clear-cut criterion to limit the number of components considered when performing LDA-PCA. In general, the number of PCs is taken to account for most of the variance. In addition, looking at the loading plots for each PC, one can notice that the curves became “noisy”, meaning that we had very rapid changes in the values by changing the wavenumbers. However, in our case, 12 components explain more than 99% of the variance, and the PC12 loading plot (Figure S9) does not show such “noisy behavior”.

It is worth mentioning that the figures of merit reported in this study are higher as compared with those published in a previous study on RCC using three supervised algorithms (k-nearest neighbors algorithm, naïve Bayes, and Random forest), performed on a reduced number of patients (23 RCC patients and 27 controls) [24].

#### 3.3.3. LDA-PCA for the Discrimination between Stages

**Figure 5:**
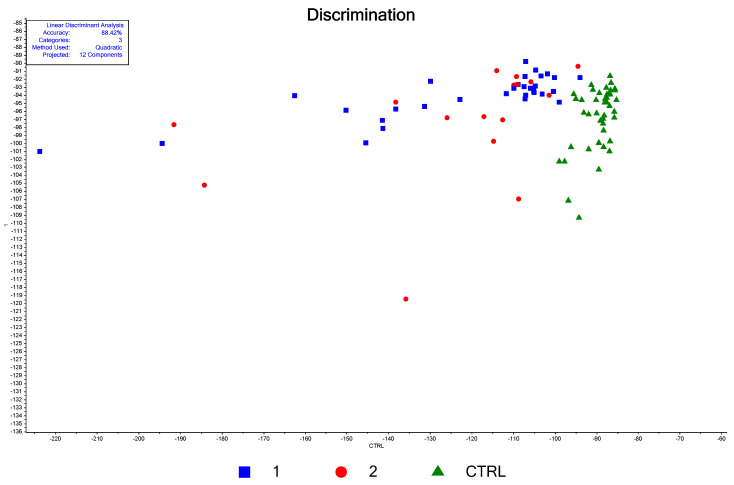
Discrimination plot for stages of RCC: green triangles = controls (CTRL), blue squares = Stage one (1), and red circles Stages 2 or 3 (for convenience in the graph legend they are marked 2).

We further checked the capability of the model to distinguish between different stages of the RCC patients based on the histopathological data (Table S1). Unfortunately, 42 of 50 patients were diagnosed with stage 1, 11 with stage 2, and 7 with stage 3. Because the number of samples should be larger than the number of components, we grouped the data for stage 2 and stage 3 (18 patients) and applied the model to discriminate between these three groups (CTRL, stage 1, and stage 2 + 3). The confusion matrix for the discrimination using the PCA-LDA model is presented in Table 4, and the discrimination plot can be found in Figure 5.

Of 45 controls, 42 were correctly classified, 2 were assigned to stage 1, and 1 was assigned to stage 2 or 3. Out of the 32 patients from the stage 1 RCC, 26 were correctly classified, 2 were assigned to the control group, and 4 were to stages 2 or 3. From the stage 2 or 3 groups, 16 were correctly classified, 1 was false negative, and 1 was assigned to stage 1. The overall accuracy of the classification was 88, i.e., 42% (84 samples correctly assigned out of 95 samples). It is quite obvious that the classification into more groups will lead to a decrease in classification accuracy, but we consider that our analysis yielded good accuracy.

#### 3.3.4. Multivariate Analysis for Normalized Data

**Figure 6:**
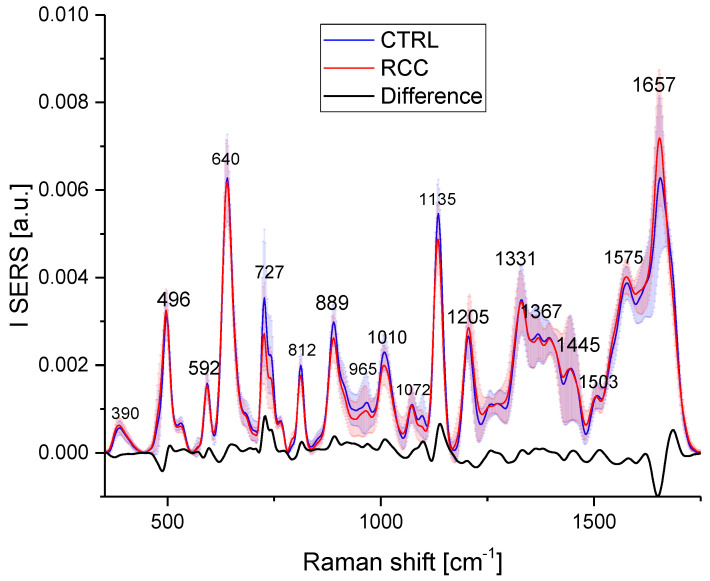
Mean SERS spectra for RCC patients samples and controls after area normalization and their difference. Shadows show the standard deviations.

As pointed out earlier, based on the comparison between the two sets of raw data, it is quite obvious that there is a statistically significant difference between the SERS intensities at almost all wavenumbers, the SERS signal from the RCC patients being more intense. All the calculations made up to this point were performed on raw data, without any preprocessing step (except the baseline correction provided by the Raman spectrometer software). We also tried to see how preprocessing can change the discrimination between the samples. Firstly, we checked to smooth the spectra using the Savitzky-Golay algorithm, but we did not observe any change in the discrimination figures. Secondly, we normalized the spectra based on three different algorithms, namely unit vector normalization, standard normal variate (SNV), and area normalization. In Figure 6 we represent the mean spectra from RCC patients and controls after area normalization and the difference between the two groups.

As expected, the normalization process reduces dramatically the differences between the controls and RCC patients (Figure 6). As a general observation, at low wave numbers (<700 cm^−1^), the differences between the two groups are insignificant; in the 700–1200 cm^−1^ the SERS intensity is higher in the control group, while above 1200 cm^−1^ the situation is reversed. Depending on the normalization procedure, the relative intensities of different peaks change dramatically, and the univariate analysis becomes meaningless. However, these quantitative changes remain significant in the context of MVA. We checked the effects of different normalization procedures on discrimination accuracy using the PCA-LDA algorithm. The best discrimination was obtained for the area normalization method (Table S4) and the quadratic discrimination function, with an overall accuracy of 98%, a sensitivity of 100%, and a specificity of 96%. These figures decrease as we pass from the quadratic function to Mahalanobis and linear discrimination function, with an overall accuracy of 92% for the latter. These results clearly show that SERS combined with MVA is indeed a very powerful tool to discriminate cancer samples from apparently healthy donors. Moreover, analyzing the loadings plot for the first two components from PCA of area normalized data (Figures S10 and S11), we see that the major vibrations contributing to PC1 are at 640 cm^−1^ (uric acid), 727 cm^−1^ (hypoxanthine), 1657 cm^−1^ (amid I proteins and eventually uric acid), and 1445 cm^−1^ (unassigned). As compared to the PCA of raw data, for which the major contributions to PC1 were the vibrations assigned to uric acid, in the case of normalized data, several vibrations belonging to different molecules contribute to PC1, and a similar observation can be made for PC2.

#### 3.3.5. SVM

Recently, it was claimed that Supported Vector Machine classification can lead to better discrimination in the LF-SERS of RCC [25], as compared to LDA-PCA. Support Vector Machine (SVM) classification, along with methods like LDA, is widely employed in data mining applications as a pattern recognition technique. We applied the SVM algorithm to our data within the Unscrambler^®^ software, which is based on the code developed by Chang and Lin [51]. The linear kernel is the simplest one, in the case of SVM, and therefore less susceptible to overfitting. For this reason, this type of kernel was used for our data analysis. In the C-type SVM, a capacity factor/penalty constant *C* should be set. For the optimization of choosing the C value, a grid method was used, within which C was varied to optimize the accuracy. We used a coarse grid search first, followed by a “fine” grid search. Another important issue that is not clearly stated in some published papers is related to the cross-validation method. We used a 10-segment cross-validation method. In 10-fold cross-validation, the program divides the training set into 10 subsets of equal size. Sequentially, one subset is tested using the classifier trained on the remaining 9 subsets. Thus, each instance of the whole training set is predicted once the cross-validation accuracy is the percentage of data that are correctly classified. The cross-validation procedure can prevent the overfitting problem. Using these parameters, the SVM algorithm using a linear kernel function has been applied to the raw data. It produced a classification with 100% accuracy for the training set and 92% for the validation set. The optimum capacity constant C was 21.54, and the number of support vectors was 22. A similar discrimination accuracy was obtained for area-normalized data, with an accuracy of 100% for the training set and 91% for the validation set, using 21 support vectors and a capacity constant C of 3.6 × 10^5^. Because the SVM method does not limit the number of samples in a set, we applied it to classify the samples according to the stage. The results (Table 5) show an overall accuracy of 81%, with all the control (45 samples) and stage 1 (32 samples) correctly classified. However, all 11 samples of stage 2 were classified as stage 1, and 5 out of 7 samples of stage 3 were classified as stage 1, 1 as stage 2, and 1 as a control. We believe that the misclassification of stages 2 and 3 is related to their significantly reduced number of samples as compared to the other groups.

Our findings indicate that the Principal Component Analysis-Linear Discriminant Analysis (PCA-LDA) offers better discrimination scores in comparison to the Support Vector Machine (SVM). While the differences may seem minimal, they are still meaningful.

In addition to superior discrimination scores, PCA-LDA also reveals the components contributing most significantly to the distinction between groups. This information, in conjunction with a detailed assignment of the spectral features, can provide valuable insights into the specific metabolites driving the discrimination. This can be of great significance for understanding the biochemical basis of the differences among the groups and can lead to more nuanced interpretations and insights.

On the other hand, SVM does allow for the identification of supporting vectors, but correlating these with the Surface Enhanced Raman Scattering (SERS) spectra poses a greater challenge. The process of linking the supporting vectors with the SERS spectra is more complex and less intuitive compared to PCA-LDA, which presents a clear-cut connection between the data and the components responsible for discrimination. Thus, from an interpretability perspective, PCA-LDA appears to be more advantageous for this study. Overall, our data show that label-free SERS combined with chemometrics allows for the identification and analysis of specific molecular signatures associated with cancer in general and with RCC in particular. Chemometrics plays a crucial role in processing and interpreting the vast amount of spectral data generated by label-free SERS measurements, enabling the extraction of relevant information from complex SERS spectra and improving the accuracy and reliability of cancer detection. Many papers were recently published in this field [52]. The integration of label-free SERS with chemometrics offers several advantages for cancer diagnosis. It eliminates the need for exogenous labels or dyes, simplifying the detection process and reducing potential interference. Additionally, the combination of SERS and chemometrics allows for high sensitivity, specificity, and multiplexing capabilities, enabling the simultaneous detection of multiple cancer biomarkers. One important limitation of our study is related to the fact that we used a state-of-the-art Raman spectrometer. For a true clinical translation of this method, one should consider this kind of high-accuracy discrimination, obtainable from a low-cost Raman device, usable at the point of care.

Nevertheless, other biological fluids like urine, saliva, and tears could be investigated for their potential use in this kind of disease. Additionally, in the case of kidney cancer, the LF-SERS approach could be successfully applied to both urine and serum, as it was recently demonstrated for other kidney diseases [53].

The application of label-free Surface Enhanced Raman Scattering (SERS) of blood components for cancer differentiation is yet to be adopted in routine clinical practice. This is primarily due to the need for more data to distinguish between various types of cancer. There are only a limited number of studies in the literature using SERS for discriminating different types of cancer (e.g., [21,25]). The translation of this technique into clinical practice requires the establishment of distinctive SERS signatures for different cancer types. Different cancers can release different biomarkers into the blood, and these need to be accurately identified and classified by the SERS technique. Without a comprehensive library of cancer-specific SERS spectra, it is challenging to reliably distinguish between cancer types.

Additionally, it is essential to consider that the biological variability among patients could lead to variations in the SERS spectra. This adds another layer of complexity to the task of distinguishing different cancer types based on SERS data. Hence, while label-free SERS of blood components holds great promise for cancer discrimination, there is still a need for more extensive data collection and research to fully exploit its potential in routine clinical practice for distinguishing between different types of cancer.

## 4. Conclusions

We utilized SERS analysis of blood serum on a solid substrate to generate highly intense and reproducible SERS spectra of biological fluids. By employing two MVA techniques, namely LDA-PCA and SVM, we successfully achieved accurate discrimination between 50 RCC serum samples and 45 controls.

With the LDA-PCA approach, we attained a remarkable discrimination accuracy of 100% by utilizing 12 principal components and a quadratic discrimination function. This result demonstrates the effectiveness of LDA-PCA in distinguishing between RCC patients and control subjects. When it comes to differentiating between different stages of RCC, the LDA-PCA approach achieved an accuracy of 88%. While this accuracy is relatively high, it indicates a slight decrease compared to the overall discrimination between RCC and controls.

Employing the SVM approach, we achieved a training accuracy of 100%, indicating that the SVM algorithm successfully learned from the training data and achieved perfect classification within that dataset. During validation, the SVM approach maintained a high accuracy of 92% when discriminating between RCC and controls. However, when it came to discriminating between different stages of RCC using the SVM approach, the accuracy obtained was 81%. This indicates a slightly lower performance in distinguishing between the various stages of RCC compared to its overall accuracy in discriminating between RCC and controls. This decrease is presumably related to the reduced number of RCC cases in stages 2 and 3.

Moreover, the main components that lead to accurate discrimination between the samples (coming from healthy and stage-diagnosed cancer patients) are the uric acid vibrational bands. These results are in agreement with recent studies showing that serum uric acid levels are positively associated with the risk of kidney cancer. Combining SERS with metabolomics analysis methods to measure the concentration of small molecular mass compounds, potential biomarkers for various diseases, would indeed be of great relevance for the SERS band assignments and for future medical applications of SERS.

Our study provides compelling evidence for the potential of combining SERS analysis with MVA techniques like LDA-PCA and SVM to effectively differentiate between RCC serum samples and controls. The utilization of label-free SERS on blood components, coupled with chemometrics, offers a promising avenue for non-invasive and early cancer detection. This approach has gained significant attention in recent years, as evidenced by the growing number of research papers focusing on its applications. The SERS technique, with its ability to detect even trace amounts of substances, cost-effectiveness, and rapid analysis, holds the potential to revolutionize cancer diagnosis across various types of cancers.

## Figures and Tables

**Table 1:** Main vibration peaks for the SERS of serum samples and their assignments using a 785 nm laser excitation line and the solid substrate.

Raman Shift Measured [cm^−1^]	Raman Shift Range Reported [cm^−1^]	Assignments in Literature	Tentative Assignments in this Study
390	382	Uric acid [22]	Uric acid
496	482–496	ν(S–S) [28] and ring vibration of L-arginine [29,30], guanine [30,31], ergothioneine [32], DNA [33]	480 Ergothioneine 496 Uric acid
593	589–592	amide-VI [30], glycerol [31] uric acid [20]	Uric acid
640	637–650	ν(C–S) of tyrosine [28,30,31,33,34,35] τ(C–C) of tyrosine [29] and phenylalanine [36], uric acid [20,22]	Uric acid
727	720–725	Hypoxanthine [20,22,29,31,37,38], δ(CH) of adenine [30,36]	Hypoxanthine
765	755–757	Tryptophan [34,35]	Uric Acid
812	813–818	ν(C–C–O) of L-serine [28,30,31], ν(C–C) of collagen [34], gluthatione [30], uric acid [20,22]	Uric acid
889	885–890	ν(C–O–H) of D-galactosamine [28,29,31,33,34,37], glutathione [30,34], uric acid [20,22]	Uric acid
1008	1002–1003	ν(C–C) in ring breathing of phenylalanine [20,29,31,34,35,37] uric acid [22]	Phenylalanine
1070	1068–1074	ν(C–C) of lipids [29,31], ν(C–N) [30] of collagen	
1135	1131–1135	ν(C–N) of D-mannose [28,29,31,36], tyrosine [20], uric acid [22]	Uric acid
1205	1205–1219	ν(C–C6H5) of tryptophan and phenylalanine rings [29,31,34,36], uric acid [22], ergothioneine [32]	Ergothioneine/Uric acid
1260	1250–1257	Amid III [34,37]	Amid III
1331	1324–1338	Adenine [28], δ(CH2) [34], hypoxanthine [38], ν(CH) of nucleic acid bases [30,31]	
1396	1400–1402	δ(CH2) of collagen [28], phospholipids [28,29], citrate [37]	
1445	1444–1450	Collagen [34,36], phospholipids [34,36], hypoxanthine [33,38], δ(CH2/CH3) [20,22,35,38] ergothioneine [32]	
1575	1576–1585	δ(C=C) of phenylalanine [28,30,33,34,36], acetoacetate [34,36,38], riboflavin [30,34,35], DNA/RNA bases [29], uric acid [20], amide II [20] hypoxanthine [22] ergothioneine [32]	Amide II
1657	1640–1680	ν(C=O) of amide I with the α-helix conformation [20,28,29,34,35,36,39] or collagen [30,31]	Amid I α helix
2107	2108	Thiocyanate [40]	Thiocyanate

ν—stretching, δ—bending, τ—twisting.

**Table 2:** Coefficient of determination for the linear correlation analysis of the SERS intensities at 640 cm^−1^ and 727 cm^−1^ with other vibration bands.

Wavenumber (cm^−1^)	Coefficient of Determination for the Linear Regression with 640 cm^−1^ (R^2^)	Wavenumber (cm^−1^)	Coefficient of Determination for the Linear Regression with 727 cm^−1^ (R^2^)
496	R^2^ = 0.93	496	R^2^ = 0.11
390	R^2^ = 0.84	390	R^2^ = 0.26
531	R^2^ = 0.80	531	R^2^ = 0.01
590	R^2^ = 0.98	590	R^2^ = 0.11
640	1	640	R^2^ = 0.15
727	R^2^ = 0.15	727	1
765	R^2^ = 0.94	765	R^2^ = 0.00
812	R^2^ = 0.91	812	R^2^ = 0.20
889	R^2^ = 0.93	889	R^2^ = 0.21
1008	R^2^ = 0.82	1008	R^2^ = 0.13
1031	R^2^ = 0.55	1031	R^2^ = 0.01
1070	R^2^ = 0.79	1070	R^2^ = 0.06
1135	R^2^ = 0.93	1135	R^2^ = 0.20
1170	R^2^ = 0.00	1170	R^2^ = 0.02
1260	R^2^ = 0.40	1260	R^2^ = 0.13
1331	R^2^ = 0.45	1331	R^2^ = 0.44
1390	R^2^ = 0.60	1390	R^2^ = 0.29
1445	R^2^ = 0.02	1445	R^2^ = 0.25
1503	R^2^ = 0.83	1503	R^2^ = 0.22
1575	R^2^ = 0.72	1575	R^2^ = 0.23
1657	R^2^ = 0.89	1657	R^2^ = 0.12

Highlights: dark gray R^2^ = 1; medium gray 0.9 < R^2^ < 1; light gray 0.8 < R^2^ < 0.9.

**Table 3:** Statistical differences between the controls and the RCC samples for the main SERS vibration bands.

Wavenumber [cm^−1^]	*p*	Interpretation
390	*p* < 0.001	Highly significant
496	*p* < 0.001	Highly significant
590	*p* < 0.001	Highly significant
640	*p* < 0.001	Highly significant
727	*p* = 0.111	Statistically insignificant
812	*p* = 0.022	Significant
889	*p* = 0.015	Significant
1008	*p* < 0.001	Highly significant
1070	*p* = 0.0012	Significant
1135	*p* = 0.039	Significant
1205	*p* < 0.001	Highly significant
1331	*p* < 0.001	Highly significant
1445	*p* = 0.016	Significant
1575	*p* < 0.001	Highly significant
1657	*p* < 0.001	Highly significant

Dark gray: highly significant; light gray: significant.

**Table 4:** Confusion matrix for the discrimination of RCC stages using the LDA-PCA model with 12 PCs.

Actual/Predicted	CTRL	Stage 1	Stages 2 and 3	Total Predicted
CTRL	42	2	1	45
Stage 1	2	26	1	29
Stages 2 and 3	1	4	16	21
Total Actual	45	32	18	

**Table 5:** Confusion matrix for the discrimination of RCC stages using the SVM model with a linear kernel.

Actual/Predicted	CTRL	Stage 1	Stage 2	Stage 3	Total Predicted
CTRL	45	0	0	1	46
Stage 1	0	32	11	5	48
Stage 2	0	0	0	1	1
Stage 3	0	0	0	0	0
Total Actual	45	32	11	7	

## Data Availability

The data sets generated and/or analyzed during the current study are available from the corresponding author upon reasonable request.

## Supplementary Materials

The following supporting information can be downloaded at: https://www.mdpi.com/article/10.3390/bios13080813/s1, Figure S1: (A) SERS spectra of all samples in the 353–2533 cm^−1^ spectral range, (B) Mean SERS spectra obtained from two different maps, Figure S2: Coefficients of determination (R2) for the correlations between the SERS intensity measured at 640 cm^−1^, assigned to uric acid, and other most intense SERS vibration bands in the serum spectra, Figure S3: Coefficients of determination (R2) for the correlations between the SERS intensity measured at 728 cm^−1^, assigned to hypoxanthine, and other most intense SERS vibration bands in the serum spectra, Figure S4: The coefficients of determination (R^2^) for the linear correlation between the SERS intensity measured at 484 cm^−1^, allegedly assigned to ergothioneine, and other SERS bands, Figure S5: Serum sample SERS in the range 442–534 cm^−1^, Figure S6: Mean SERS intensities comparison between the controls and RCC samples for two wavenumbers: 640 cm^−1^ and 728 cm^−1^, Figure S7: Explained variance for PCA with 12 principal components, Figure S8 Loading plot for PC2, Figure S9: Loading plot for PC12,; Figure S10: Loadings plot of the first component (PC1) showing the contribution of the variables to this component, within the PCA, applied for area normalized data, Figure S11: Loadings plot of the second component (PC2) showing the contribution of the variables to this component, within the PCA, applied for area normalized data, Table S1: Demographic data and tumor-related information of the renal cell carcinoma patients enrolled in the study, Table S2: Demographic data of control patients, Table S3: The coefficients of determination (R^2^) for the correlations between the SERS intensity measured at 484 cm^−1^, allegedly assigned to ergothioneine, and other SERS vibration assigned to the same compound, Table S4: Accuracy of discrimination of RCC samples for different preprocessing steps, using LDA-PCA with 12 components, Table S5 Accuracy of discrimination of RCC samples for different preprocessing steps, using LDA-PCA with 12 components.
