# Supplementary material for: High-Accuracy Renal Cell Carcinoma Discrimination through Label-Free SERS of Blood Serum and Multivariate Analysis
Source: Biosensors (Basel). 2023 Aug 13;13(8):813. doi: 10.3390/bios13080813 (PMC10452371; doi:10.3390/bios13080813)

Supplementary Materials

Supplementary Table S1. Demographic data and tumor-related information of the renal cell carcinoma patients enrolled in the study.

Number	Age (years)	Sex	Tumor histology	TNM	Stage	ISUP grade	Intratumoral necrosis	Intratumoral bleeding
1	66	M	Clear cell carcinoma	T1bN0M0	1	3	Yes	No
2	76	M	Clear cell carcinoma	T3aN0M0	3	4	Yes	Yes
3	73	M	Clear cell carcinoma	T1aN0M0	1	1	No	No
4	44	M	Clear cell carcinoma	T1aN0M0	1	2	No	Yes
5	73	M	Clear cell carcinoma	T3aN0M0	3	2	No	Yes
6	62	M	Clear cell carcinoma	T1bN0M0	1	2	Yes	Yes
7	56	M	Clear cell carcinoma	T1bN0M0	1	2	No	Yes
8	64	M	Clear cell carcinoma	T1aN0M0	1	1	No	No
9	77	M	Clear cell carcinoma	T2aN0M0	2	2	No	No
10	67	M	Clear cell carcinoma	T1bN0M0	1	2	No	No
11	60	M	Clear cell carcinoma	T1aN0M0	1	2	No	Yes
12	59	M	Clear cell carcinoma	T1aN0M0	1	2	No	Yes
13	72	M	Clear cell carcinoma	T2aN0M0	2	3	Yes	Yes
14	67	M	Clear cell carcinoma	T3aN0M0	3	4	Yes	Yes
15	75	M	Clear cell carcinoma	T2aN0M0	2	2	No	Yes
16	47	M	Clear cell carcinoma	T2aN0M0	2	1	Yes	Yes
17	61	M	Clear cell carcinoma	T2aN0M0	2	2	No	Yes
18	55	M	Clear cell carcinoma	T1aN0M0	1	2	No	Yes
19	61	M	Clear cell carcinoma	T1aN0M0	1	2	No	No
20	64	M	Clear cell carcinoma	T3aN0M0	3	2	Yes	Yes

21	54	M	Clear cell carcinoma	T1aN0M0	1	2	No	Yes
22	62	M	Clear cell carcinoma	T1bN0M0	1	4	Yes	Yes
23	53	M	Clear cell carcinoma	T2aN0M0	2	2	No	Yes
24	60	M	Clear cell carcinoma	T2aN0M0	2	3	No	Yes
25	64	M	Clear cell carcinoma	T1bN0M0	1	1	Yes	Yes
26	60	M	Clear cell carcinoma	T2aN0M0	2	1	No	Yes
27	44	M	Clear cell carcinoma	T1bN0M0	1	3	No	No
28	66	M	Clear cell carcinoma	T1aN0M0	1	1	No	Yes
29	38	M	Clear cell carcinoma	T1aN0M0	1	1	No	No
30	59	M	Clear cell carcinoma	T3aN0M0	3	2	Yes	Yes
31	69	M	Clear cell carcinoma	T1bN0M0	1	2	No	Yes
32	74	M	Clear cell carcinoma	T1bN0M0	1	2	Yes	Yes
33	76	M	Clear cell carcinoma	T2aN0M0	2	2	Yes	Yes
34	72	M	Clear cell carcinoma	T1aN0M0	1	2	No	Yes
35	70	M	Clear cell carcinoma	T1bN0M0	1	1	No	Yes
36	69	M	Clear cell carcinoma	T1bN0M0	1	1	Yes	Yes
37	57	M	Clear cell carcinoma	T1aN0M0	1	2	No	No
38	56	M	Clear cell carcinoma	T1bN0M0	1	1	No	Yes
39	63	M	Clear cell carcinoma	T2aN0M0	2	2	No	Yes
40	78	M	Clear cell carcinoma	T1aN0M0	1	2	No	Yes
41	64	M	Clear cell carcinoma	T3aN0M0	3	4	Yes	Yes
42	65	M	Clear cell carcinoma	T1aN0M0	1	2	No	Yes
43	56	M	Clear cell carcinoma	T3aN0M0	3	2	Yes	Yes
44	70	M	Clear cell carcinoma	T1aN0M0	1	2	No	Yes

45	50	M	Clear cell carcinoma	T1aN0M0	1	2	No	No
46	69	M	Clear cell carcinoma	T1aN0M0	1	2	No	Yes
47	41	M	Clear cell carcinoma	T1aN0M0	1	1	No	No
48	54	M	Clear cell carcinoma	T1bN0M0	1	4	Yes	Yes
49	48	M	Clear cell carcinoma	T1aN0M0	1	2	No	Yes
50	68	M	Clear cell carcinoma	T2aN0M0	2	2	No	Yes

Supplementary Table S2. Demographic data of control patients.

Number	Age (Years)	Sex
1	40	M
2	57	M
3	64	M
4	79	M
5	62	M
6	48	M
7	68	M
8	68	M
9	74	M
10	60	M
11	48	M
12	38	M
13	84	M
14	53	M
15	73	M
16	59	M
17	66	M
18	19	M
19	31	M
20	72	M
21	74	M
22	19	M
23	83	M
24	45	M
25	73	M
26	54	M
27	60	M
28	65	M
29	82	M
30	68	M
31	50	M

32	60	M
33	74	M
34	73	M
35	88	M
36	62	M
37	52	M
38	70	M
39	66	M
40	62	M
41	26	M
42	64	M
43	59	M
44	51	M
45	53	M

Figure S1. SERS spectra of A: all samples in the 353-2533 cm^{-1} spectral range, patients (RCC blue) and control (CTRL in red); B mean SERS intensities for the two maps

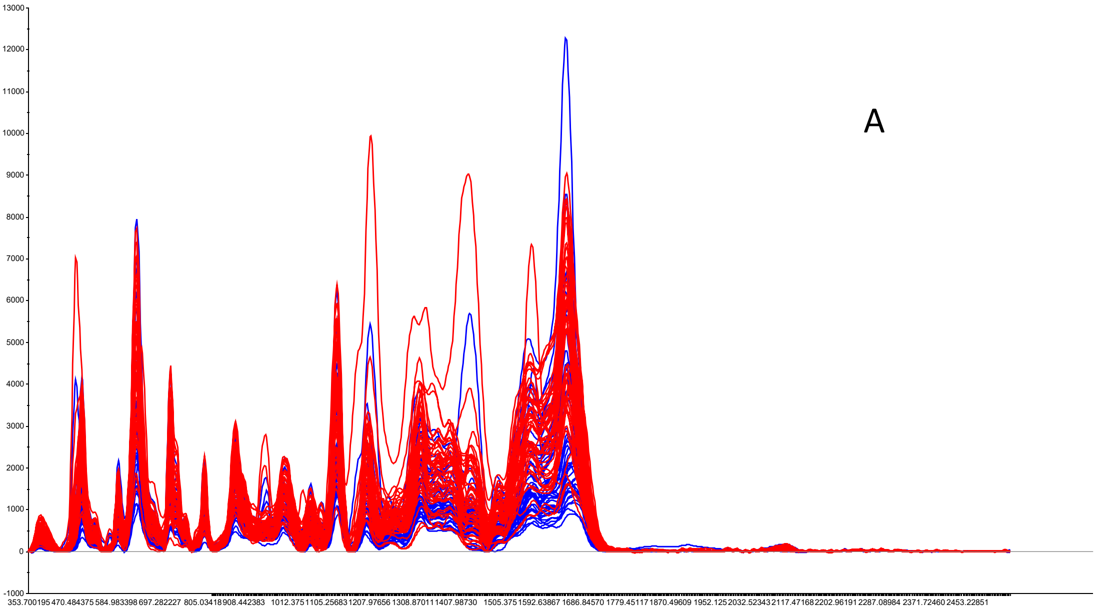

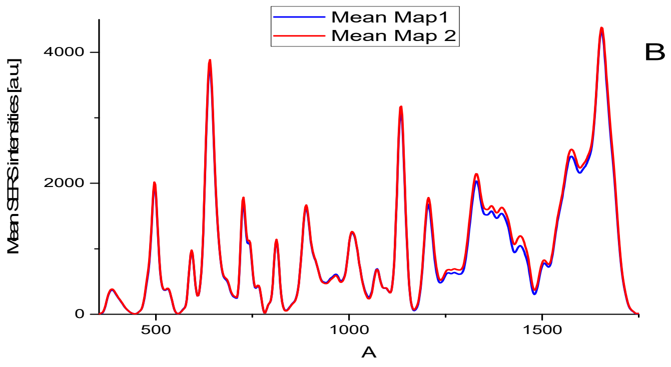

Figure S2. Coefficients of determination (R^2) for the correlations between the SERS intensity measured at 640 cm^{-1}, assigned to uric acid, and other most intense SERS vibration bands in the serum spectra.

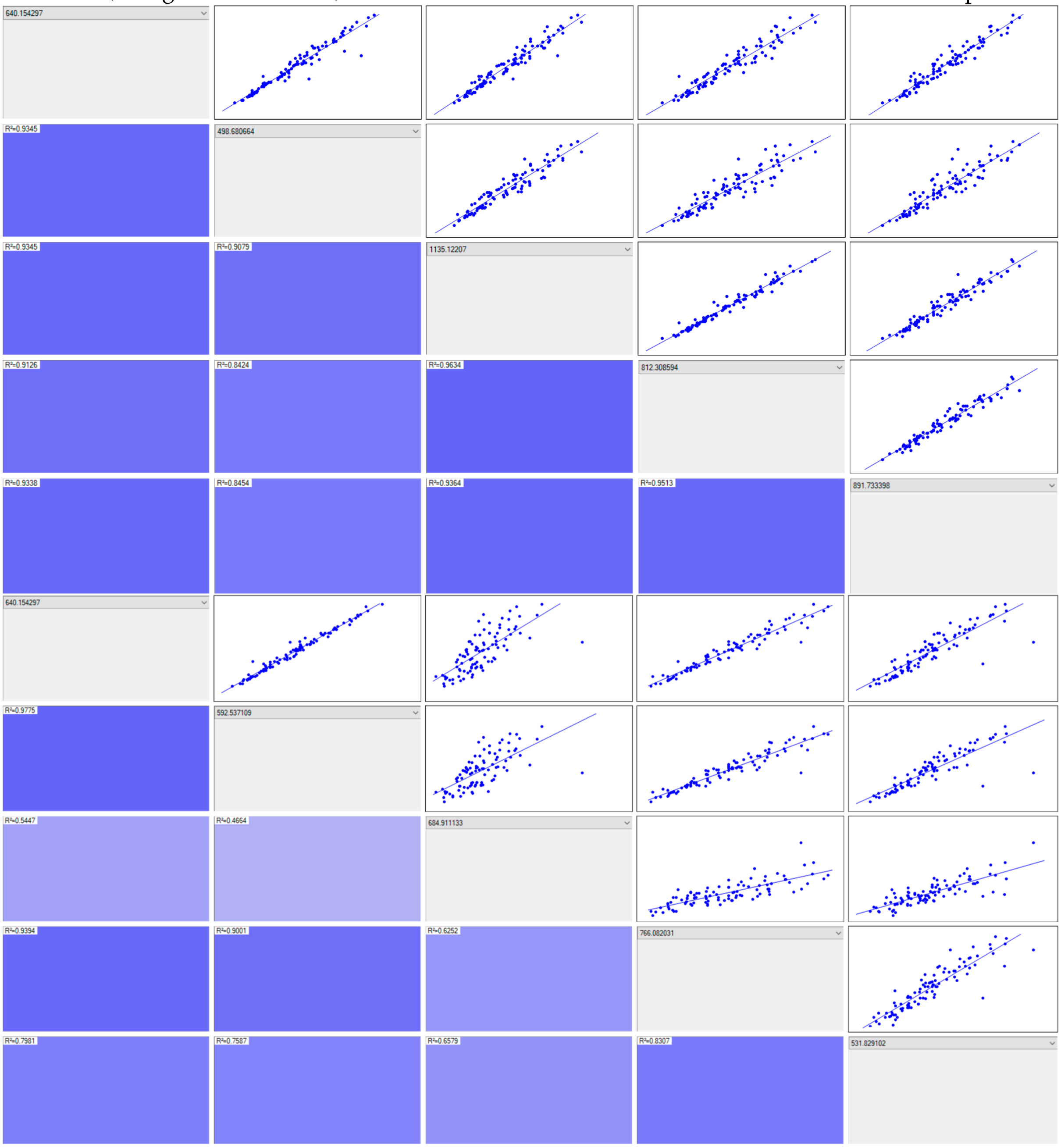

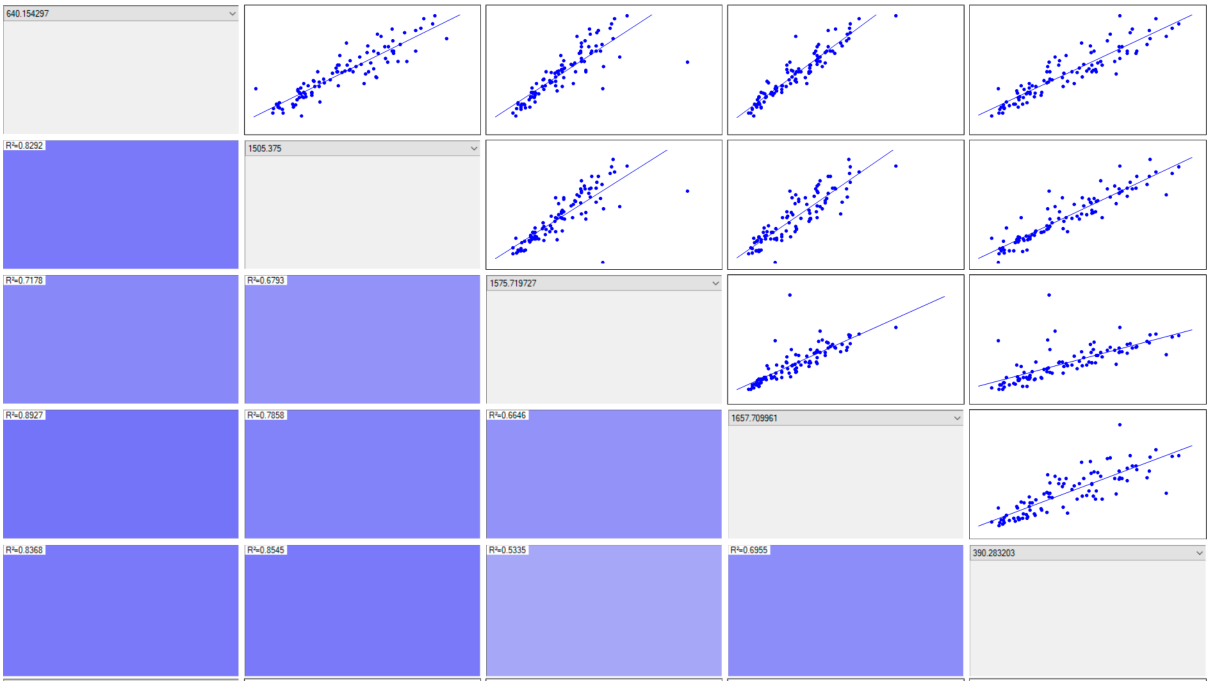
640.154297
R^2=0.8292
1505.375
R^2=0.7178
R^2=0.6793
1575.719727
R^2=0.8927
R^2=0.7858
R^2=0.6646
1657.709961
R^2=0.8368
R^2=0.8545
R^2=0.5335
R^2=0.6955
390.283203

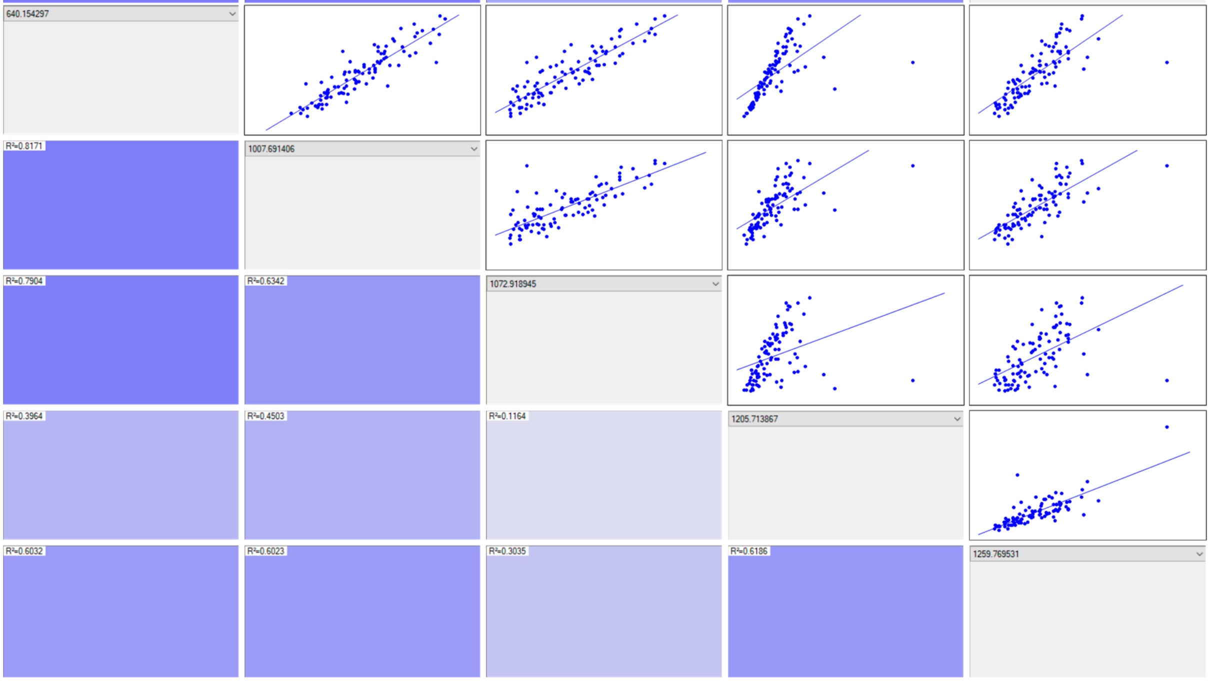
640.154297
R^2=0.8171
1007.691406
R^2=0.7904
R^2=0.6342
1072.918945
R^2=0.3964
R^2=0.4503
R^2=0.1164
1205.713867
R^2=0.6032
R^2=0.6023
R^2=0.3035
R^2=0.6186
1259.769531

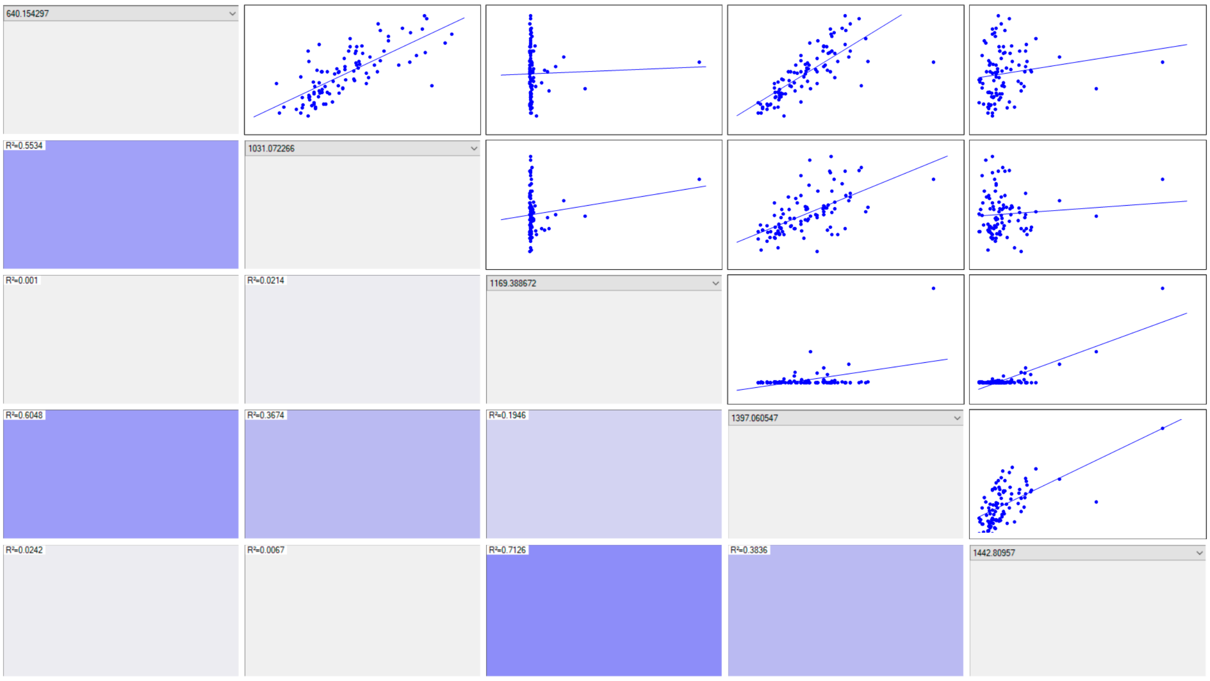

Figure S3. Coefficients of determination (R^2) for the correlations between the SERS intensity measured at 727 cm^{-1}, assigned to hypoxanthine, and other most intense SERS vibration bands in the serum spectra.

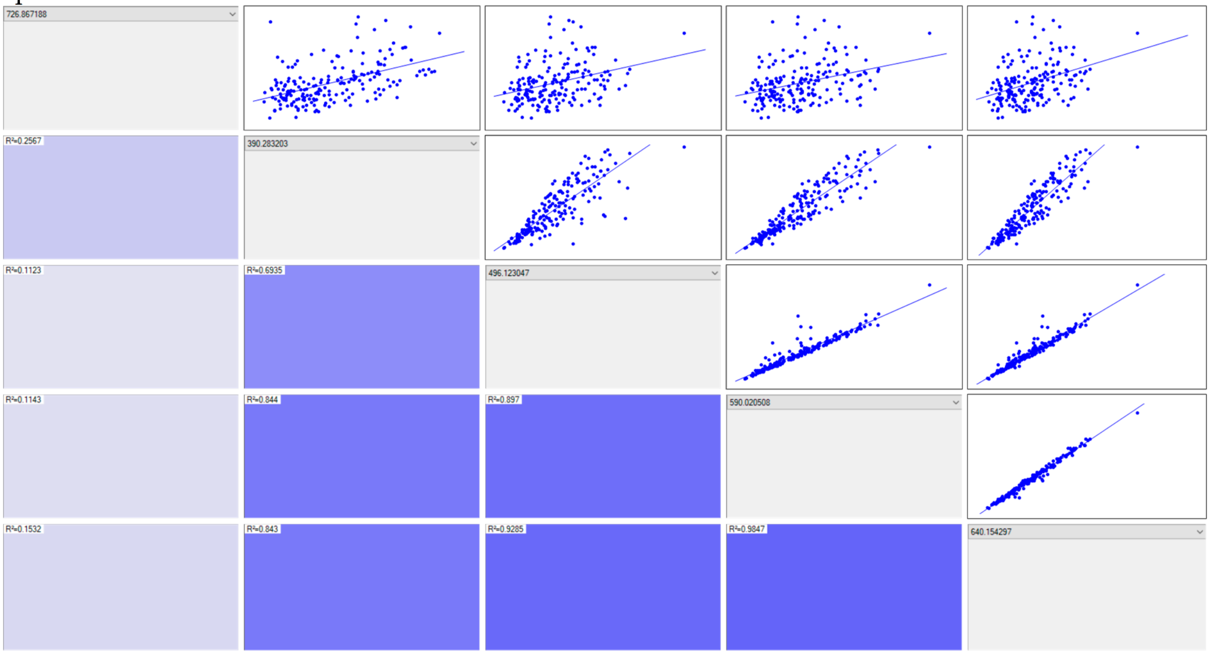

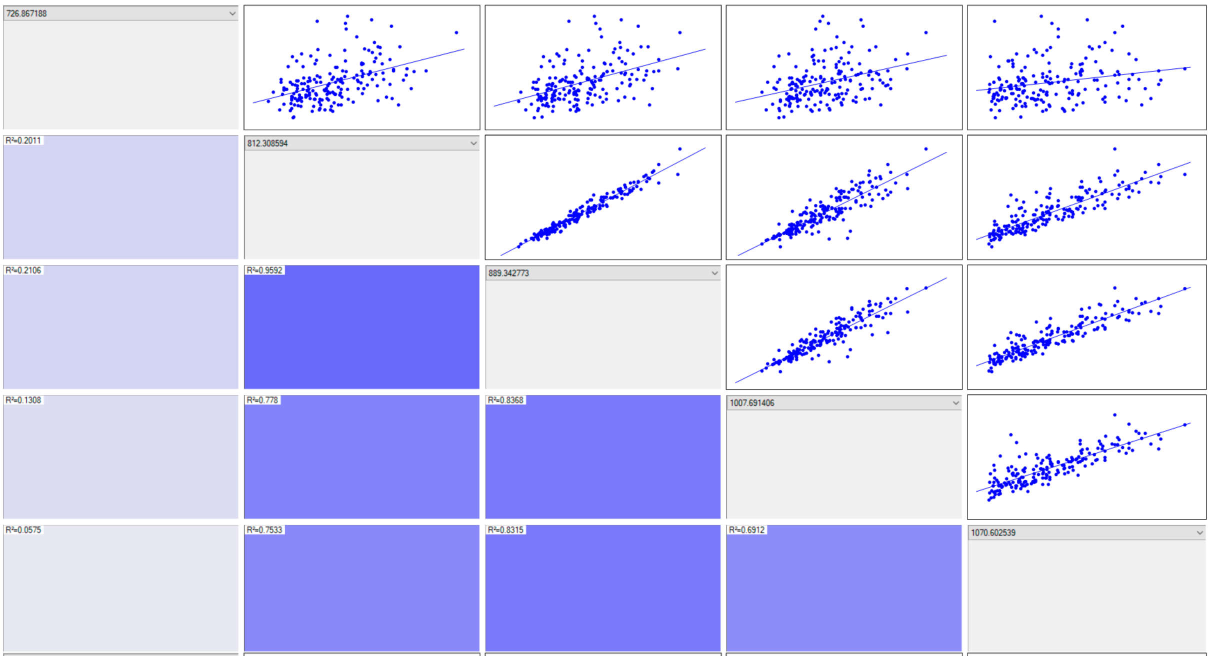
726.867188
R^2=0.2011
812.308594
R^2=0.2106
R^2=0.9592
889.342773
R^2=0.1308
R^2=0.778
R^2=0.8368
1007.691406
R^2=0.0575
R^2=0.7533
R^2=0.8315
R^2=0.6912
1070.602539

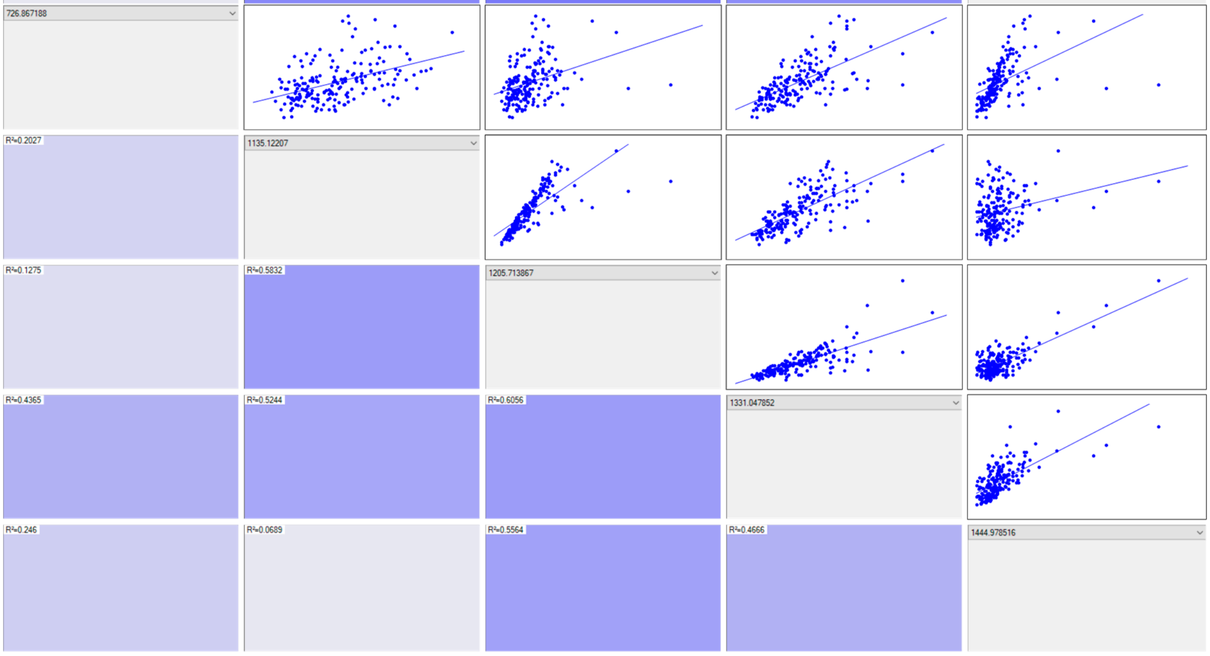
726.867188
R^2=0.2027
1135.12207
R^2=0.1275
R^2=0.5832
1205.713867
R^2=0.4365
R^2=0.5244
R^2=0.6056
1331.047852
R^2=0.246
R^2=0.0689
R^2=0.5564
R^2=0.4666
1444.978516

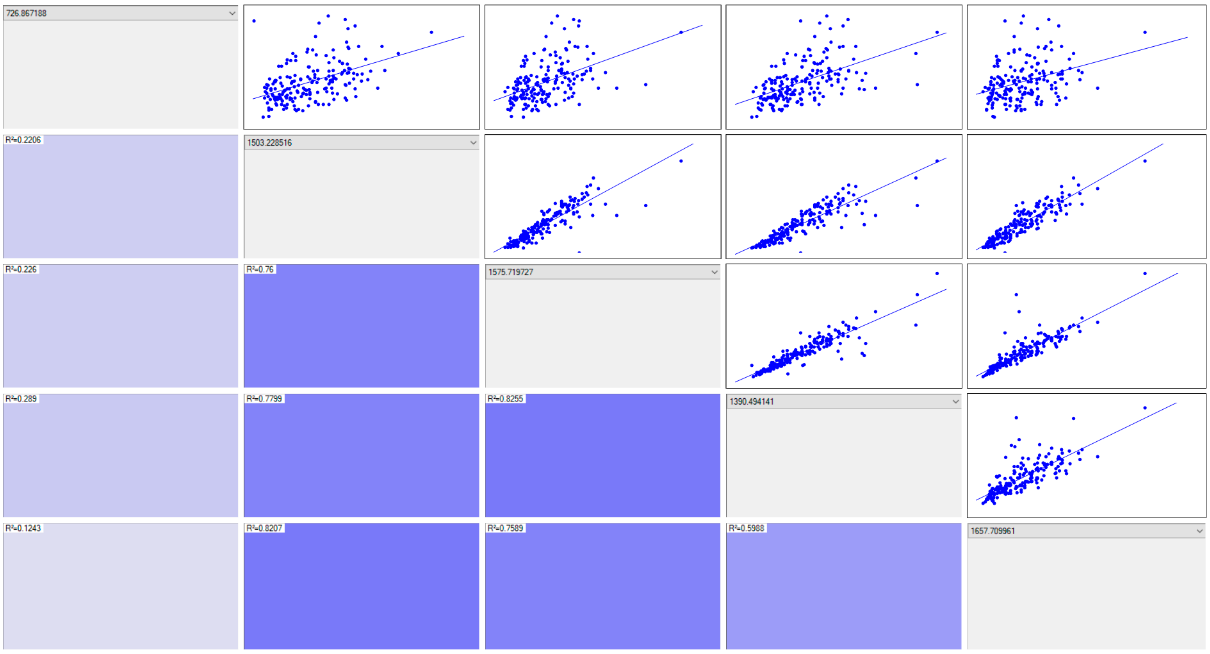

Figure S4. The coefficients of determination (R^2) for the correlations between the SERS intensity measured at 484 cm^{-1}, allegedly assigned to ergothioneine, and other SERS vibration assigned to the same compound.

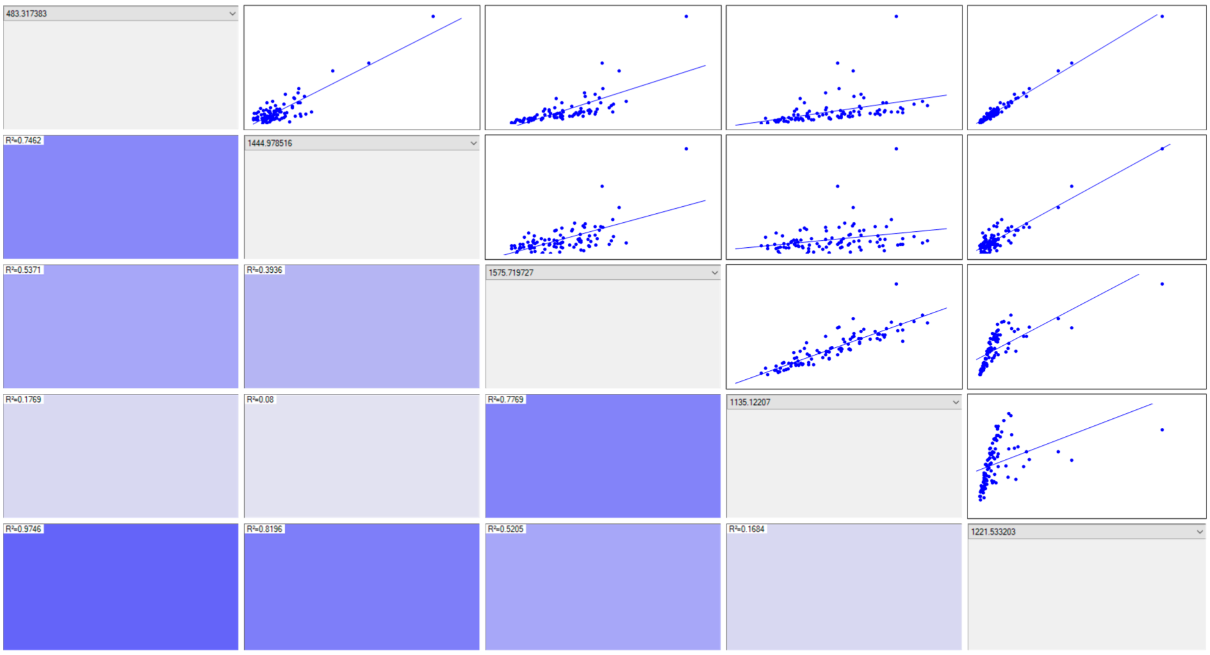

Table S3. The coefficients of determination (R^2) for the correlations between the SERS intensity measured at 484 cm^{-1}, allegedly assigned to ergothioneine, and other SERS vibration assigned to the same compound.

Wavenumber (cm-1)	Coefficient of determination for the linear regression with 484 cm^{-1} (R^2)
1444.98	R^2=0.7462
1575.72	R^2=0.5442
1132.83	R^2=0.2314
1214.76	R^2=0.9526

Figure S5. Serum sample SERS in the range 442-534 cm^{-1} (blue are controls and red RCC)

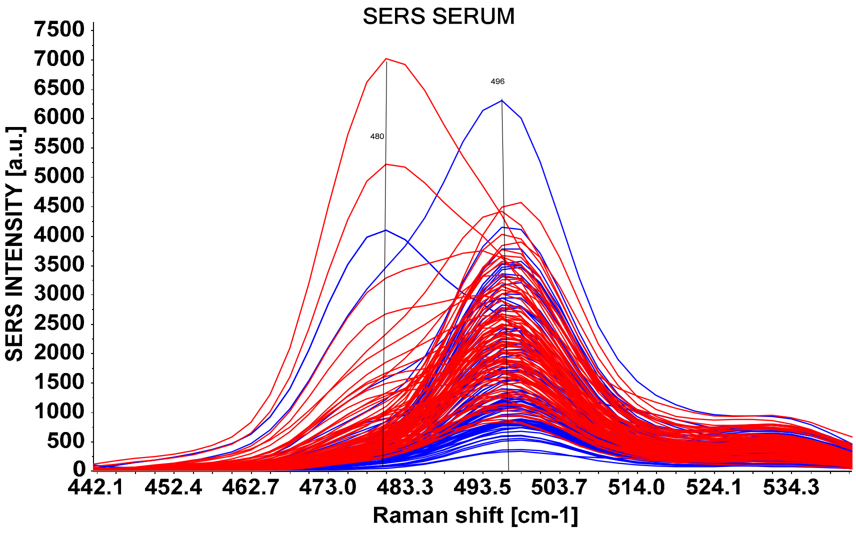

Figure S6. Mean SERS intensities comparison between the controls and RCC samples for two wavenumbers: 640 cm^{-1} and 727 cm^{-1}

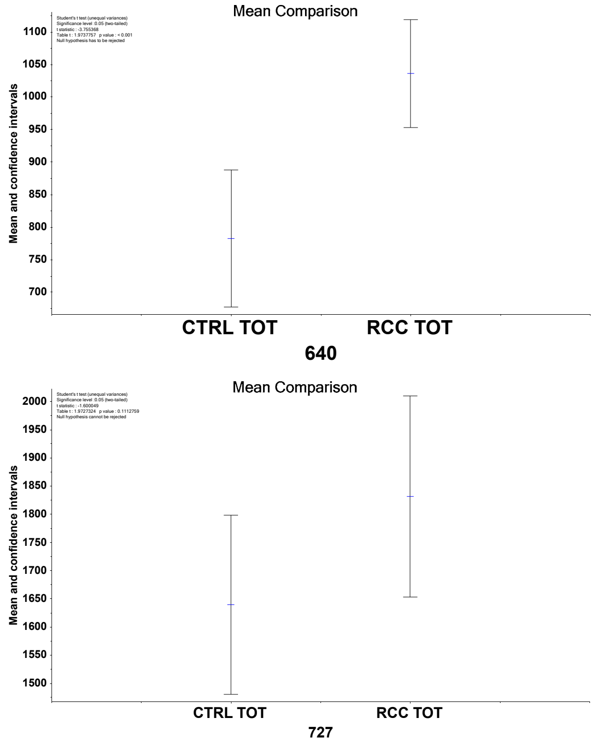

Figure S7. Explained variance for PCA with 12 principal components

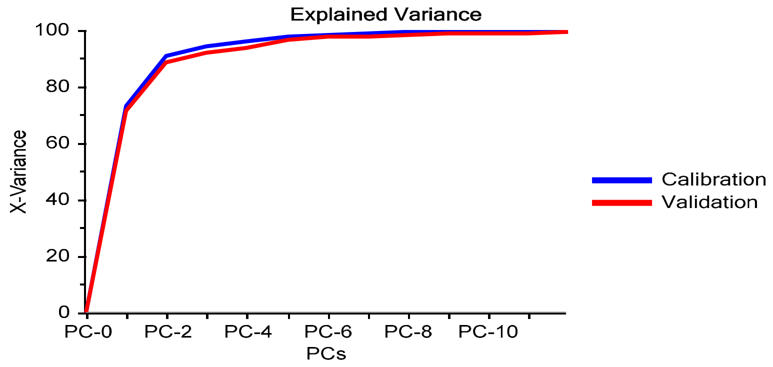

Figure S8 Loading plot for PC2 for raw data

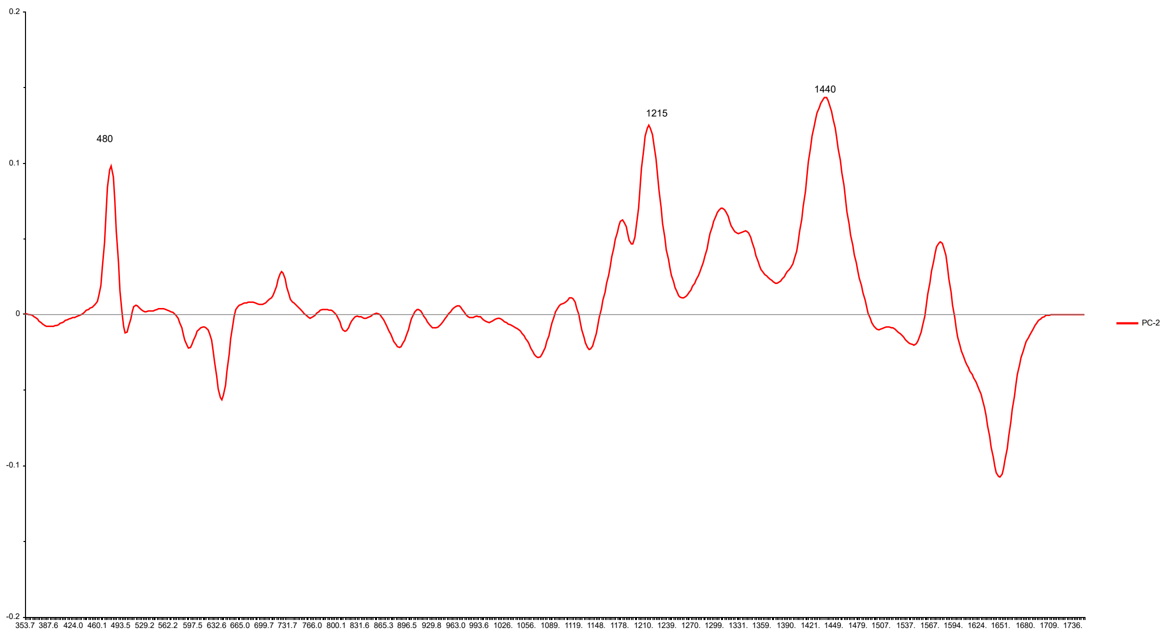

Figure S9 Loading plot for PC12 for raw data

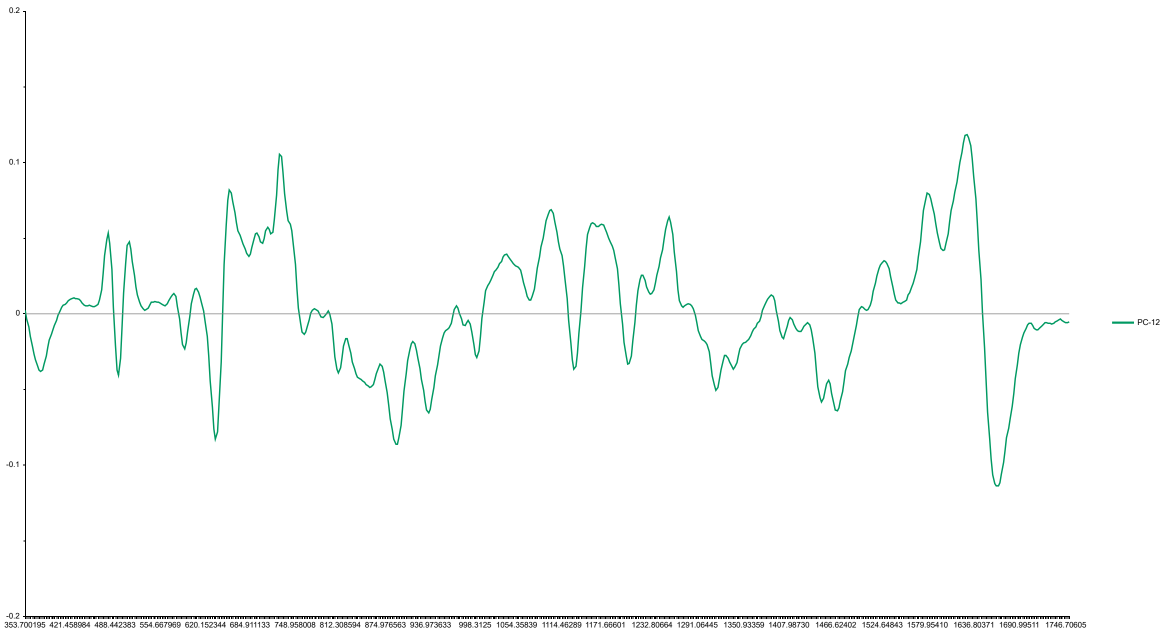

Table S4. Discrimination accuracy as a function of the number of components and the discrimination function.

No of components	Discrimination function	TP	TN	FP	FN	Sensitivity	Specificity	Accuracy
5	Linear	35	32	13	15	0.7	0.71	0.71
	Quadratic	25	40	5	25	0.5	0.89	0.68
	Mahalanobis	47	22	23	3	0.94	0.49	0.73
7	Linear	34	32	13	16	0.68	0.71	0.69
	Quadratic	41	38	7	9	0.82	0.84	0.83
	Mahalanobis	49	24	21	1	0.98	0.53	0.77
8	Linear	34	31	14	16	0.68	0.69	0.68
	Quadratic	42	40	5	8	0.84	0.89	0.86
	Mahalanobis	49	22	23	1	0.98	0.49	0.75
9	Linear	34	37	8	16	0.68	0.82	0.75
	Quadratic	46	39	6	4	0.92	0.87	0.89
	Mahalanobis	49	30	15	1	0.98	0.67	0.83
10	Linear	41	40	5	9	0.82	0.89	0.85
	Quadratic	49	42	3	1	0.98	0.93	0.96
	Mahalanobis	50	32	13	0	1	0.71	0.86
11	Linear	43	42	3	7	0.86	0.93	0.89
	Quadratic	50	43	2	0	1	0.96	0.98
	Mahalanobis	50	34	11	0	1	0.76	0.88
12	Linear	44	43	2	6	0.88	0.96	0.92
	Quadratic	50	45	0	0	1	1.00	1.00
	Mahalanobis	50	34	11	0	1	0.76	0.88

TP= true positive; TN=true negative; FP=false positive; FN=false negative; Sensitivity=TP/(TP+FN); Specificity=TN/(TN+FP); Accuracy=(TP+TN)/(TP+TN+FP+FN)

Table S5 Accuracy of discrimination of RCC samples for different preprocessing steps, using LDA-PCA with 12 components

**Preprocessing step**	**No preprocessing**	**Vector normalization**	**SNV**	**Area normalization**
Accuracy	100%	96%	96%	98%

Figure S10. Loadings plot of the first component (PC1) showing the contribution of the variables to this component, within the PCA, applied for area normalized data

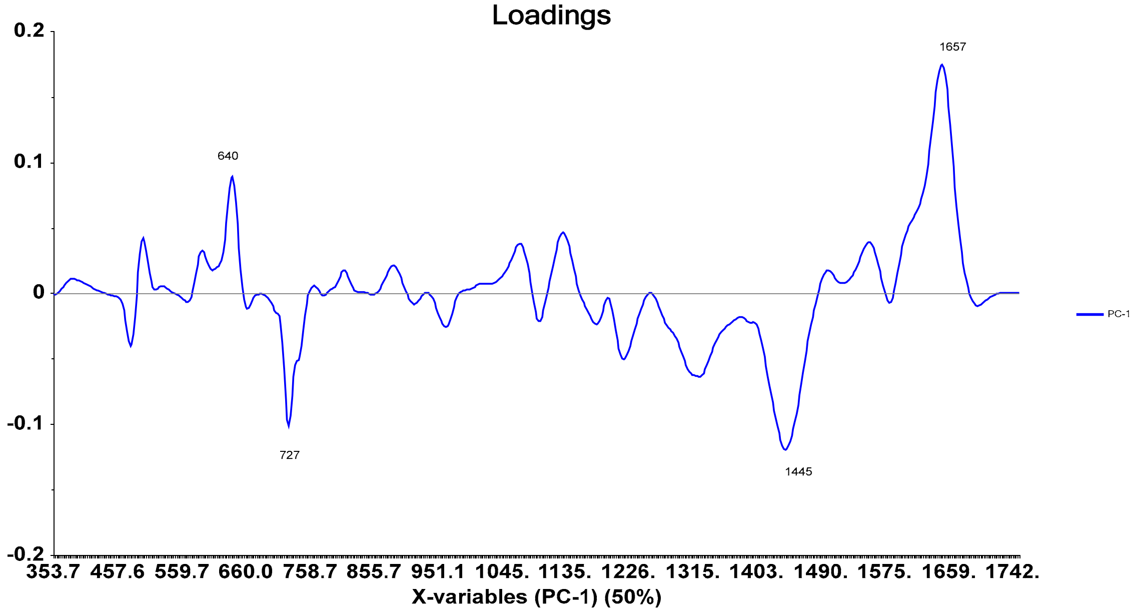

Figure S11. Loadings plot of the second component (PC2) showing the contribution of the variables to this component, within the PCA, applied for area normalized data

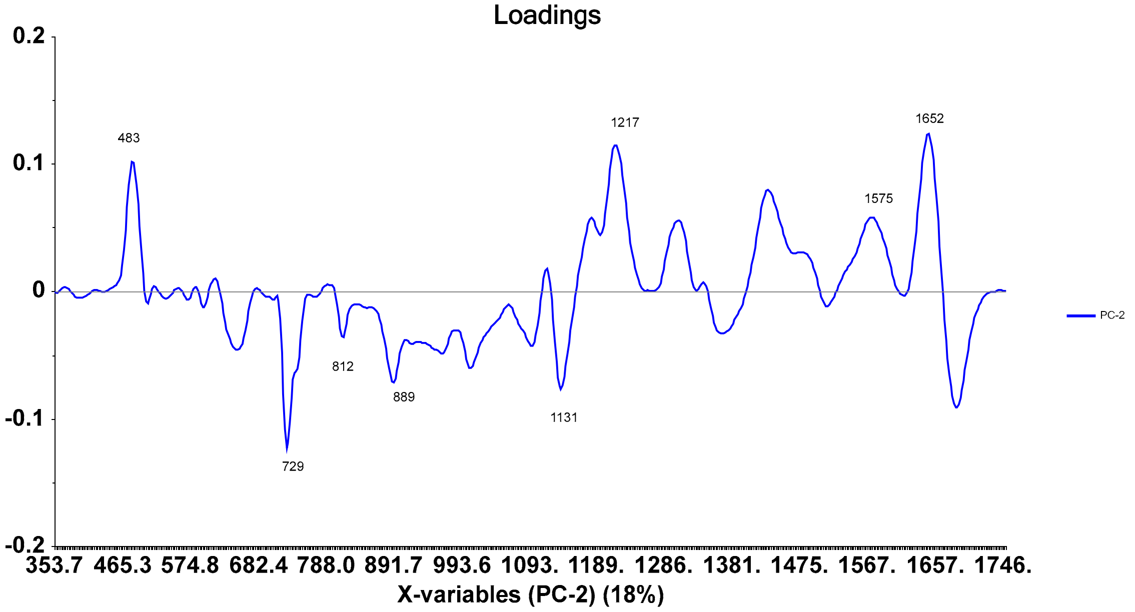

Supplement: Supplementary file 1 [file biosensors-13-00813-s001.zip › Supplementary Information Biosensors 6.pdf]
